# An endolysin gene from *Candidatus* Liberibacter asiaticus confers dual resistance to huanglongbing and citrus canker

**DOI:** 10.1093/hr/uhad159

**Published:** 2023-08-08

**Authors:** Lanzhen Xu, Kaiqing Mo, Danlu Ran, Juanjuan Ma, Lehuan Zhang, Yijia Sun, Qin Long, Guojin Jiang, Xiaochun Zhao, Xiuping Zou

**Affiliations:** Integrative Science Center of Germplasm Creation in Western China (CHONGQING) Science City, Citrus Research Institute, Southwest University/National Citrus Engineering Research Center, Chongqing 400712, China; Integrative Science Center of Germplasm Creation in Western China (CHONGQING) Science City, Citrus Research Institute, Southwest University/National Citrus Engineering Research Center, Chongqing 400712, China; Integrative Science Center of Germplasm Creation in Western China (CHONGQING) Science City, Citrus Research Institute, Southwest University/National Citrus Engineering Research Center, Chongqing 400712, China; Integrative Science Center of Germplasm Creation in Western China (CHONGQING) Science City, Citrus Research Institute, Southwest University/National Citrus Engineering Research Center, Chongqing 400712, China; Integrative Science Center of Germplasm Creation in Western China (CHONGQING) Science City, Citrus Research Institute, Southwest University/National Citrus Engineering Research Center, Chongqing 400712, China; Integrative Science Center of Germplasm Creation in Western China (CHONGQING) Science City, Citrus Research Institute, Southwest University/National Citrus Engineering Research Center, Chongqing 400712, China; Integrative Science Center of Germplasm Creation in Western China (CHONGQING) Science City, Citrus Research Institute, Southwest University/National Citrus Engineering Research Center, Chongqing 400712, China; Integrative Science Center of Germplasm Creation in Western China (CHONGQING) Science City, Citrus Research Institute, Southwest University/National Citrus Engineering Research Center, Chongqing 400712, China; Integrative Science Center of Germplasm Creation in Western China (CHONGQING) Science City, Citrus Research Institute, Southwest University/National Citrus Engineering Research Center, Chongqing 400712, China; Integrative Science Center of Germplasm Creation in Western China (CHONGQING) Science City, Citrus Research Institute, Southwest University/National Citrus Engineering Research Center, Chongqing 400712, China

## Abstract

The most damaging citrus diseases are Huanglongbing (HLB) and citrus canker, which are caused by *Candidatus* Liberibacter asiaticus (*Ca*Las) and *Xanthomonas citri* pv. *citri* (*Xcc*), respectively. Endolysins from bacteriophages are a possible option for disease resistance in plant breeding. Here, we report improvement of citrus resistance to HLB and citrus canker using the LasLYS1 and LasLYS2 endolysins from *Ca*Las. LasLYS2 demonstrated bactericidal efficacy against several Rhizobiaceae bacteria and *Xcc*, according to inhibition zone analyses. The two genes, driven by a strong promoter from *Cauliflower mosaic virus*, 35S, were integrated into Carrizo citrange via *Agrobacterium*-mediated transformation. More than 2 years of greenhouse testing indicated that *LasLYS2* provided substantial and long-lasting resistance to HLB, allowing transgenic plants to retain low *Ca*Las titers and no obvious symptoms while also clearing *Ca*Las from infected plants in the long term. *LasLYS2* transgenic plants with improved HLB resistance also showed resistance to *Xcc*, indicating that LasLYS2 had dual resistance to HLB and citrus canker. A microbiome study of transgenic plants revealed that the endolysins repressed Xanthomonadaceae and Rhizobiaceae populations in roots while increasing Burkholderiaceae and Rhodanobacteraceae populations, which might boost the citrus defense response, according to transcriptome analysis. We also found that Lyz domain 2 is the key bactericidal motif of LasLYS1 and LasLYS2. Four endolysins with potential resistance to HLB and citrus canker were found based on the structures of LasLYS1 and LasLYS2. Overall, the work shed light on the mechanisms of resistance of *Ca*Las-derived endolysins, providing insights for designing endolysins to develop broad-spectrum disease resistance in citrus.

## Introduction

Citrus huanglongbing (HLB), which occurs frequently in all major citrus-growing regions in the world, is undoubtedly the world’s most devastating citrus disease. HLB is caused by *Candidatus* Liberibacter asiaticus (*Ca*Las), which belongs to the phloem-limited, unculturable, fastidious Alphaproteobacteria, and its vector in the field is the Asian citrus psyllid (*Diaphorina citri*) [1]. HLB causes billions of dollars of losses to the citrus industry every year [2]. Thus far, there are no cures available to citrus farmers. Furthermore, citrus canker, induced by *Xanthomonas citri* pv. *citri* (*Xcc*), is another severe bacterial disease of citrus in the world. Especially in recent years, it has shown an outbreak trend, mainly because many eradication programs are too costly to continue for a long time [3, 4], which is imposing additional pressure on the citrus industry threatened by HLB. Replacement of susceptible cultivars with broad-spectrum resistant ones is the most efficient strategy to control HLB and citrus canker. However, to date, no HLB resistance has been identified in the genus *Citrus*, and almost all citrus cultivars are susceptible to citrus canker [2, 4, 5]. Thus, improving disease resistance has been an important citrus breeding objective. Citrus improvement using conventional breeding approaches is difficult and time-consuming due to factors inherent in citrus, such as the long juvenile period, incompatibility, heterozygosity, and polyembryony [6]. Genetic engineering of existing citrus cultivars is the fastest and most economical method for establishing resistance to HLB and citrus canker and has been used in several citrus improvement programs [7–10].

Endolysins produced by bacteriophages have been extensively used for pathogen control in humans and animals [11, 12]. Endolysins lyse bacterial cell walls to release progeny phages, resulting in a sudden drop in turgor pressure and osmotic lysis and thus killing the host [12]. Compared with conventional broad-spectrum antibiotics, the major advantage of endolysins is their higher specificity, although they may also show effects against different bacterial species. Certain endolysins only kill a highly specific set of bacterial species or strains and do not harm humans or animal cells [13]. Even so, the lytic spectrum of an endolysin could be changed using molecular engineering [14, 15]. Meanwhile, endolysins have rapid bactericidal activity by lysing bacterial cells in minutes or even seconds [13]. Moreover, endolysin-resistant bacteria have rarely been reported to date, due to the importance and conservation of the peptidoglycan layer, which is the main target of endolysins, and to the anti-biofilm activity of endolysin [12, 16]. Thus, endolysin is believed to be one of the best alternatives for pathogen control in the agricultural industry [17].

There have been a few cases of a bacteriophage-encoded endolysin being used to boost disease resistance in plants. Ectopic production of *Propionibacterium* phage P1.1 endolysin LysP11 in tobacco demonstrated significant antibacterial action against *Erysipelothrix rhusiopathiae* [18]. CMP1, an endolysin from the *Clavibacter michiganensis* subsp. *michiganensis* phage CMP1, also conferred resistance to *C. michiganensis* in transgenic tomatoes [19]. Bacteriophages have been effectively utilized in the field to reduce citrus canker and citrus bacterial spot [20], showing that endolysins may be beneficial for engineering resistance to bacterial diseases in citrus.

The *Ca*Las genome carries two prophages, SC1 and SC2, which were predicted to have a lytic and lysogenic cycle, respectively [21]. Activation of *Ca*Las prophage or its lytic genes is believed to negatively correlate with *Ca*Las pathogenicity or infection [22, 23], suggesting that *Ca*Las prophages and their lytic genes are a powerful means to control HLB by triggering bacterial ‘suicide’ to suppress *Ca*Las pathogenicity. There are two predicted endolysin genes, *LasLYS1* (CLIBASIA_04790) and *LasLYS2* (CLIBASIA_04800), in the *Ca*Las genome [21], but their functions are still undetermined. In this study, we showed that ectopic expression of *LasLYS1* and *LasLYS2* in Carrizo citrange (*Citrus sinensis* × *Poncirus trifoliata*) exhibited enhanced resistance to HLB. LasLYS2 has strong and dual resistance to HLB and citrus canker and can completely inhibit *Ca*Las bacteria from colonizing transgenic plants. We also evaluated the structure of the bacterial community and plant immune responses affected by *LasLYS1* and *LasLYS2* expression in roots and midribs, where plants directly challenge *Ca*Las attack, and highlighted the important characteristics associated with protein structure for engineering endolysins and promoting broad-spectrum disease resistance in citrus.

## Results

### LasLYS1 and LasLYS2 have selective bactericidal activity

**Figure 1:**
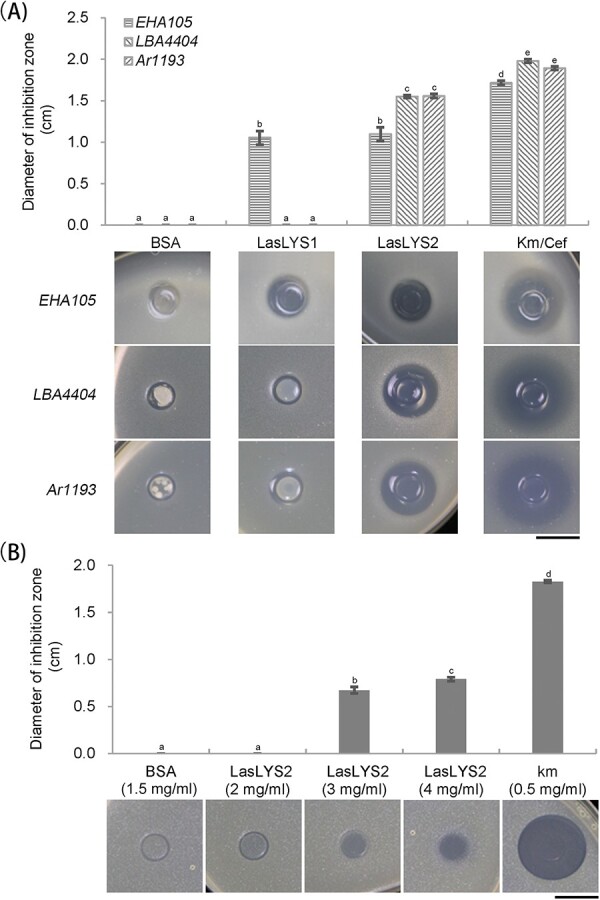
Antibacterial activities of LasLYS1 and LasLYS2 recombinant proteins, using BSA and kanamycin (Km) or cefotaxime (Cef) as negative and positive controls, respectively. (A) Antibacterial activities of LasLYS1 and LasLYS2 against *Agrobacterium* EHA105, LBA4404, and Ar1193 strains. A total of 50 μl solution containing 1.5 mg/ml BSA, 1.5 mg/ml LasLYS1, 1.5 mg/ml LasLYS2, or 0.5 mg/ml kanamycin (for the Ar1193 strain, 0.5 mg/ml cefotaxime was used as a positive control) was added into the Oxford cup. (B) Antibacterial activity of LasLYS2 against *X. citri* pv. *citri*. After 3 days of inoculation, the size of the inhibition zone was measured. Values are expressed as means ± standard deviation of three independent tests. Different letters at the top of the bars represent significant differences from the BSA control based on Duncan’s test (*P* < .05). Scale bar = 1 cm.

The bactericidal activity of LasLYS1 and LasLYS2 against a panel of selected bacterial strains was determined *in vitro* using the Oxford cup method*. Ca*Las belongs to the Rhizobiaceae [24]. Thus, the selected bacterial strains mainly included Rhizobiaceae (*A. tumefaciens* and *A. rhizogenes*) species. The citrus canker pathogen *Xcc* was also tested. Because *Ca*Las is an unculturable bacterium, it was not possible to include it in this test. *LasLYS1* and *LasLYS2* were expressed in *Escherichia coli* cells for harvesting the proteins. The bactericidal activities of different concentrations of recombinant proteins were first tested against *A. tumefaciens* EHA105 and *Bacillus thuringiensis* HD73 strains. Both LasLYS1 and LasLYS2 at 1.5 mg/ml concentration can induce visible inhibition zones against EHA105 (Supplementary Data Fig. S1). Lower concentrations (0.5 and 1.0 mg/ml) of proteins did not induce a visible inhibition zone (Supplementary Data Fig. S1). These concentrations of recombinant proteins had no activity against HD73 (Supplementary Data Fig. S1). Thus, we further investigated the bactericidal activity of 1.5 mg/ml recombinant proteins against 10 other bacteria using 0.5 mg/ml kanamycin and 5 mg/ml bovine serum albumin (BSA) as positive and negative controls, respectively (Supplementary Data Fig. S2). LasLYS2 produced inhibition zones of ~1.5 cm for *A. rhizogenes* Ar1193 and *A. tumefaciens* LBA4404 compared with ~1.9 cm for the kanamycin control (Fig. 1A and Supplementary Data Fig. S2). No distinguishable inhibition zones were induced by LasLYS2 against *A. rhizogenes* strains ArQual, MSU4404, K599, and C58C1, or *A. tumefaciens* strains GV3101, AGL1, and EHA101, or *Xcc* (Supplementary Data Fig. S2). LasLYS1 did not introduce distinguishable inhibition zones for all 10 tested bacterial strains. The data demonstrated that the endolysins have selective bactericidal activity against some Rhizobiaceae strains, and LasLYS2 has more broad-spectrum bactericidal activity than LasLYS1.

The results showed that the *Xcc* colonies in the Oxford cups supplied with 1.5 mg/ml LasLYS1 and LasLYS2 were thinner than those in the cups treated with 1.5 mg/ml BSA (Supplementary Data Fig. S2). This suggests that LasLYS1 and LasLYS2 should have antibacterial action against *Xcc*. As a result, we examined the antibacterial efficacy of recombinant proteins at increasing doses against *Xcc*. LasLYS1 at 2, 3, and 4 mg/ml did not exhibit any discernible antibacterial action (Supplementary Data Fig. S2). LasLYS2 did, however, exhibit substantial inhibitory zones at concentrations of 3 and 4 mg/ml (Fig. 1B), demonstrating that it had antibacterial action against *Xcc*.

### LasLYS2 confers high resistance to HLB in transgenic Carrizo citranges

Under control of a strong promoter, 35S, plant codon-optimized *LasLYS1* and *LasLYS2* were introduced into Carrizo citrange by *Agrobacterium*-mediated epicotyl transformation (Supplementary Data Fig. S3). GUS histochemical labeling (Supplementary Data Fig. S3) and PCR analysis (Supplementary Data Fig. S4) were used to screen transgenic plants. Quantitative RT–PCR (qRT–PCR) and RT–PCR demonstrated the presence of high transgene expression levels in eight LasLYS1 and six LasLYS2 transgenic plants (Supplementary Data Fig. S4). Transgenic shoots, including wild-type (WT) controls, were cut into five or six parts for rooting (Supplementary Data Fig. S3). Each separate transgenic line produced three to five rooted plants on average. In the greenhouse, no change in plant shape or growth was found between transgenic plants and WT controls (Supplementary Data Fig. S3).

**Figure 2:**
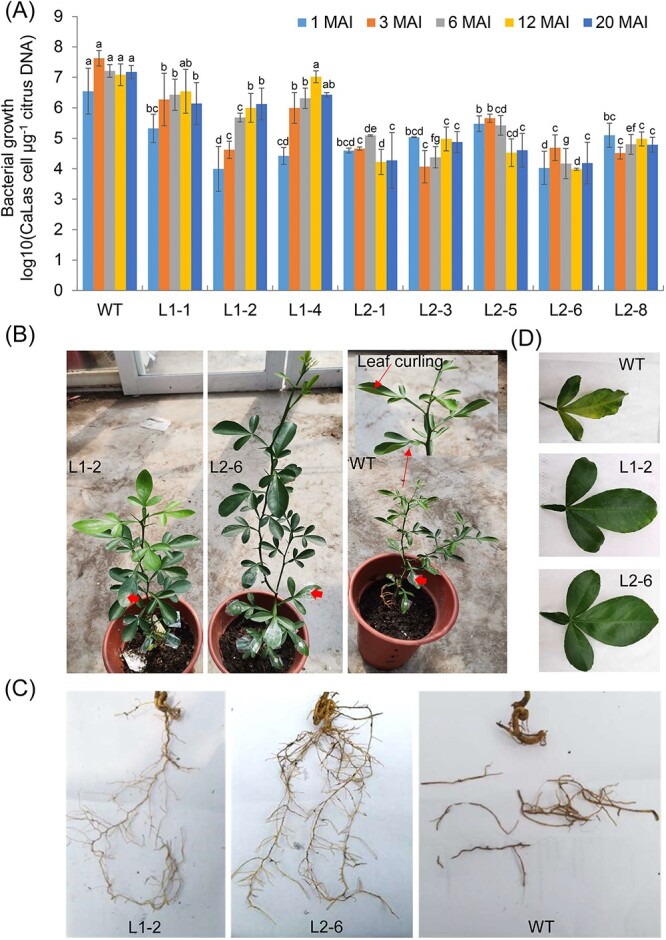
Evaluation of HLB resistance of transgenic plants in a greenhouse. (A) Characteristics of *Ca*Las growth in the transgenic plants. Bacterial populations [log_10_ (CaLas cells μg^−1^ of citrus DNA)] in roots were investigated using qPCR. Values are expressed as means ± standard deviation of three or four plants per line. Different letters at the top of the bars indicate significant differences from the WT control based on Duncan’s test (*P* < .05). (B)–(D) Comparison of HLB symptoms in transgenic plants infected by *Ca*Las in a greenhouse. Symptoms of plants (B) and roots (C) after 4 months of *Ca*Las infection. Symptoms of leaves (D) after 12 months of *Ca*Las infection. WT plants developed more severe HLB symptoms, such as leaf curling and root decline, than transgenic lines. Thick red arrows indicate infection sites grafted with leaf discs containing *Ca*Las. L1-# and L2-#, transgenic plants expressing *LasLYS1* and *LasLYS2*, respectively.

Resistance to HLB in 14 transgenic plants was determined using the method of leaf-disc grafting infection [25]. First, the growth of *Ca*Las in roots was determined by quantitative PCR (qPCR) since it was reported that *Ca*Las tends to first colonize roots [25, 26]. At 1, 3, and 6 months after infection, significantly decreased contents of *Ca*Las were detected in the *LasLYS1* lines L1-1, L1-2, L1-4, L1-7, L1-10, and L1-12, and the *LasLYS2* lines L2-1, L2-3, L2-5, L2-6, and L2-8, compared with the WT control (Fig. 2A and Supplementary Data Fig. S5). Consistently low levels of *Ca*Las were observed in the L1-2, L2-1, L2-3, L2-5, L2-6, and L2-8 lines, but not in the L1-7, L1-10, or L1-12 lines at 12 and 20 months after infection. The *Ca*Las contents in all these lines were still significantly lower than those in the WT control (Fig. 2A and Supplementary Data Fig. S5). The resistance levels of *LasLYS2* transgenic plants were more than two orders of magnitude higher than those of *LasLYS1* transgenic plants, as determined by the bacterial populations as the disease intensity index (Fig. 2A).

The L1-1, L1-2, L1-4, L2-1, L2-3, and L2-6 lines were more resistant to HLB than the other lines (Fig. 2). As a result, the six lines were chosen to explore *Ca*Las development in leaf and root tissues over extended time periods. *Ca*Las levels in leaf and root tissues from the majority of the six lines were still considerably lower than in the WT control from 25 to 29 months after infection (Table 1 and Supplementary Data Tables S1 and S2). More crucially, we found no *Ca*Las bacteria in the L2-3 or L2-6 lines’ leaf or root tissues, showing that LasLYS2 endolysin is extremely successful at clearing the *Ca*Las.

**Table 1:** Characteristics of *Ca*Las growth in representative transgenic plants.

Line	Bacterial growth [log_10_ (*Ca*Las cells μg^−1^ of citrus DNA)]					
	25 MAI		27 MAI			29 MAI
Leaf	Root	Leaf	Root	Leaf	Root	
WT	7.52 ± 0.09^a^	6.92 ± 0.03^a^	7.33 ± 0.35^a^	7.55 ± 0.53^a^	7.15 ± 0.15^a^	7.73 ± 0.62^a^
LI-1	7.17 ± 0.21^b^	6.86 ± 0.46^a^	6.46 ± 0.22^b^	6.95 ± 0.10^b^	6.76 ± 0.26^ab^	7.06 ± 0.01^b^
LI-2	7.19 ± 0.16^b^	6.44 ± 0.29^ab^	6.25 ± 0.18^bc^	6.84 ± 0.21^b^	6.32 ± 0.22^b^	7.11 ± 0.13^b^
LI-4	6.75 ± 0.05^c^	6.03 ± 0.02^b^	5.94 ± 0.15^c^	6.29 ± 0.11^c^	5.81 ± 0.18^c^	6.35 ± 0.09^c^
L2-1	5.71 ± 0.21^d^	4.58 ± 0.19^c^	4.83 ± 0.04^d^	5.32 ± 0.08^d^	4.66 ± 0.44^d^	5.21 ± 0.21^d^
L2-3	N.D.	N.D.	N.D.	N.D.	N.D.	N.D.
L2-6	N.D.	N.D.	N.D.	N.D.	N.D.	N.D.

qPCR was used to look at the bacterial populations [log_10_ (*Ca*Las cells μg^−1^ of citrus DNA)] in root and leaf tissues. Values are expressed as means ± standard deviation of three or four plants per line. Different lower-case letters after the values indicate significant differences from the WT control based on Duncan’s test (*P* < .05). L1-# and L2-#, transgenic plants expressing *LasLYS1* and *LasLYS2*, respectively. MAI, months after infection. N.D., *Ca*Las bacteria were not detected.

Some WT plants showed signs of root rot after 1 month of infection while transgenic plants did not. Three months later, all WT plants displayed symptoms of decayed and retarded roots, reduced lateral roots, and upwardly curled leaves, and some *LasLYS1* transgenic plants also displayed these symptoms (Fig. 2B and C). However, *LasLYS2* transgenic plants did not exhibit these symptoms in their roots or leaves (Fig. 2B and C). Some *LasLYS1* transgenic and WT plants showed significant chlorosis signs, such as mottled yellow leaves, after 12 months of infection (Fig. 2D). Nevertheless, over the 29-month assessment period, these symptoms were not seen on the leaves of LasLYS2 transgenic plants.

The aforementioned information taken together demonstrated that Carrizo citrange was more resistant to HLB after ectopic expression of LasLYS1 and LasLYS2, with LasLYS2-mediated resistant being greater.

### LasLYS2 confers resistance to citrus canker in transgenic Carrizo citranges

**Figure 3:**
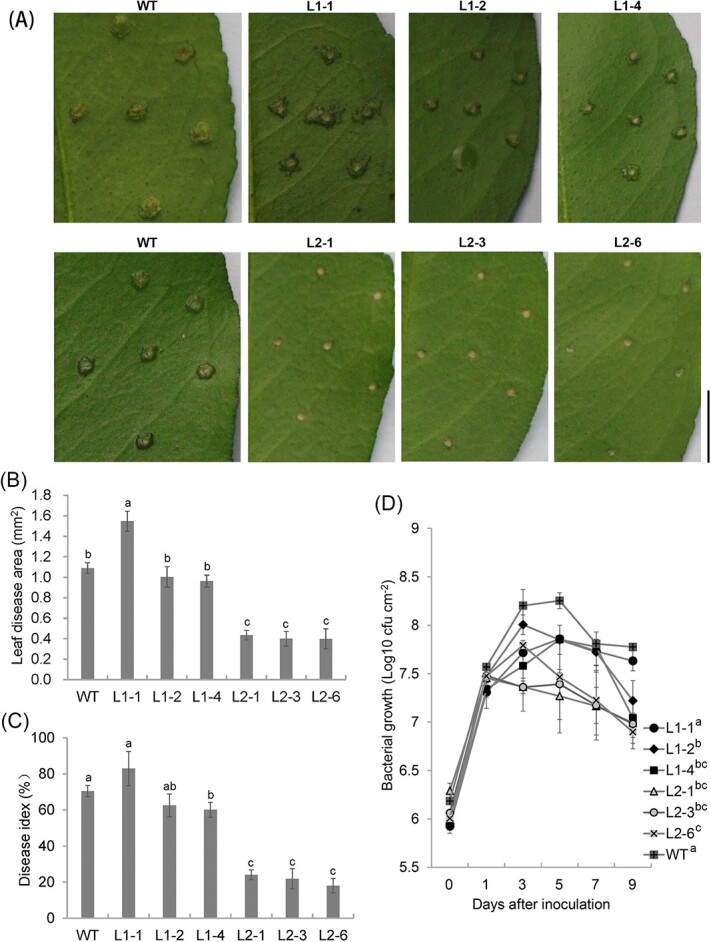
Citrus canker resistance in transgenic plants. 1 × 10^8^ CFU/ml *X. citri* pv. *citri* (*Xcc*) was injected into fully grown leaves of transgenic and WT plants. At 9 dpi, the symptoms of citrus canker (A), diseased area (B), and disease severity (C) of transgenic lines’ leaves were assessed. (D) *Xcc* growth in transgenic plant leaves. Values are expressed as means ± standard deviation of three independent tests. Different lower-case letters above the bars in (B) and (C) and next to the line designators in (D) show significant deviations from the WT control at 9 dpi according to Duncan’s test (*P* < .05). L1-# and L2-#, transgenic lines expressing *LasLYS1* and *LasLYS2*, respectively. Scale bar = 5 mm.

The Oxford cup test showed that LasLYS2 has *in vitro* antibacterial activity against *Xcc*. To further determine its antibacterial activity against *Xcc* in citrus plants, we evaluated citrus canker resistance in the L1-1, L1-2, L1-4, L2-1, L2-3, and L2-6 lines, which showed high resistance to HLB in previous experiments. *Xcc* cells were inoculated by pinprick inoculation [9]. At 9 days post-inoculation (dpi), the L1-2 and L1-4 lines showed smaller diseased lesions than those of the WT control and the L1-1 line (Fig. 3A). No visible pustules or cankers were detected in the L2-1, L2-3, or L2-6 lines (Fig. 3A), demonstrating that LasLYS2 can completely suppress the development of pustules incited by *Xcc*. The statistical analysis showed that there was no obvious difference in either disease areas or disease index between the *LasLYS1* transgenic lines and WT plants (Fig. 3B and C). However, the disease areas in the *LasLYS2* transgenic lines were 0.40–0.43 mm^2^, which was significantly smaller than that (1.09 mm^2^) of WT plants (Fig. 3B). Indeed, estimation of disease severity revealed that the disease index of L2-1 (24.0%), L2-3 (21.8%), and L2-6 (18.0%) was significantly lower than that of WT plants (70.5%) at 9 dpi (Fig. 3C). Evaluation of *Xcc* growth demonstrated a slower growth rate in all the transgenic plants tested (Fig. 3C). The *Xcc* population in all the *LasLYS2* transgenic plants was significantly smaller than that observed in WT plants, and it was also smaller than that in *LasLYS1* transgenic plants at 5 dpi (Fig. 3C). The canker resistance levels of the L2-1, L2-3, and L2-6 lines were also confirmed by *in vivo* infiltration assays (Supplementary Data Fig. S6). In these transgenic lines, canker symptoms were significantly milder compared with those in the WT control during the inoculation. These results clearly demonstrate that transgenic lines expressing *LasLYS2* possess high resistance to citrus canker.

### Characteristics of endophytic bacteria community in transgenic plants

**Figure 4:**
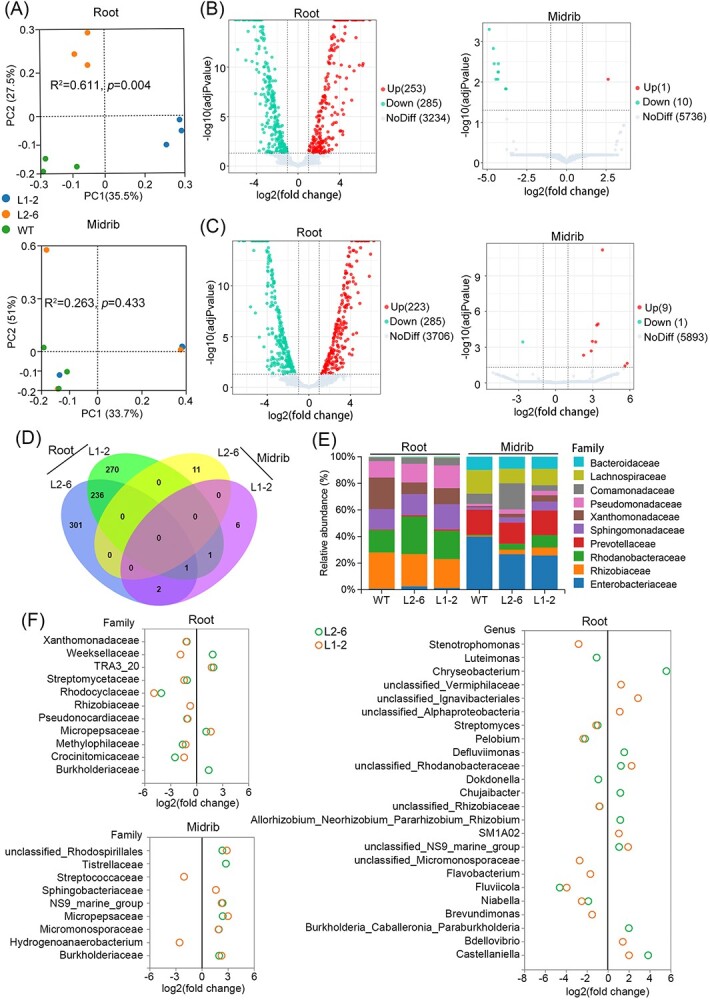
Microbiome characteristics in transgenic plant roots and midribs. (A) Comparison of root and midrib microbiomes in L1-2 and L2-6 transgenic lines with WT controls (PERMANOVA, *P* < .01, *n* = 3). The findings show that the microbiomes in transgenic roots differ significantly from those in WT roots. (B) and (C) Volcano plot showing differentially abundant ASVs in L1-2 (C) and L2-6 (B) transgenic lines compared with WT controls (Supplementary Data S2–S5). The red and blue plots show that bacterium abundance has increased (positive log_2_ fold change) and decreased (negative log_2_ fold change), respectively (adjusted *P* < .05). (D) Venn diagram illustrating the overlaps of differentially abundant ASVs between the L2-6 and L1-2 transgenic lines in comparison with WT controls. (E) Bacterial family abundance in the root and midrib microbiomes of L2-6 and L1-2 transgenic lines, compared with WT controls. (F) Differential abundance analysis of bacterial families and genera in L2-6 and L1-2 transgenic lines against WT control (adjusted *P* < .05; Supplementary Data S6 and S7). Here is an example of differentially abundant taxa with >0.1 relative abundance (%).

The Oxford cup test demonstrated the selective bactericidal activity of LasLYS1 and LasLYS2 (Fig. 1), indicating their potential impact on the endophytic bacterial community in transgenic plants. Consequently, we conducted a characterization of the microbiota in the root and midrib of healthy transgenic plants, comparing them with healthy WT controls, using 16S rRNA deep sequencing with three biological replicates. For this study, we selected the L2-6 and L1-2 lines, which exhibited high resistance to HLB. On average, each sample yielded 159 706 clean reads and 1292 amplicon sequence variants (ASVs) (Supplementary Data S1). No significant shifts were observed in the overall composition of bacterial phyla or the total microbial diversity (α-diversity) in the roots and midribs of transgenic plants compared with WT controls (Supplementary Data Fig. S7). However, principal coordinate analysis based on Bray–Curtis dissimilarities (β-diversity) revealed a noteworthy separation of root microbiomes between the L2-6, L1-2, and WT plants, while no such separation was detected in their midrib microbiomes (Fig. 4A). In the roots of L2-6 and L1-2 lines, 538 and 508 differentially abundant ASVs were identified, respectively, whereas only 11 and 10 were found in their midribs when compared with WT controls (Fig. 4B and C and Supplementary Data S2–S5). A total of 236 differentially abundant ASVs were shared by the roots of the L2-6 and L1-2 lines (Fig. 4D). These findings suggested that the ectopic expression of *LasLYS1* and *LasLYS2* significantly influences the composition of the root microbiome.

Comparison of microbial relative abundance showed that, compared with WT controls, the populations of Xanthomonadaceae and Rhizobiaceae decreased, while the populations of Comamonadaceae, Pseudonocardiaceae, Rhodanobacteraceae, and Enterobacteriaceae increased in the roots of L2-6 and L1-2 lines (Fig. 4E and Supplementary Data Fig. S7). In the midribs, the populations of Lachnospiraceae and Enterobacteriaceae decreased while those of Xanthomonadaceae and Rhodanobacteraceae increased in transgenic plants (Fig. 4E and Supplementary Data Fig. S7). We further investigated the changes in bacterial families with >0.01% relative abundance in comparison with the WT control. In the L2-6 line, 23 out of 33 significantly abundant bacterial families displayed decreased populations in roots. In midribs, 20 out of 23 significantly abundant bacterial families had increased populations (Supplementary Data S6). In the L1-2 line, the number of significantly abundant bacterial families with an increased or decreased abundance was almost equal in the roots, while 55 out of 62 significantly abundant bacterial families had an increased abundance in the midribs (Supplementary Data S6). The abundances of Xanthomonadaceae, Streptomycetaceae, Rhodocyclaceae, Pseudonocardiaceae, Methylophilaceae and Crocinitomicaceae decreased significantly in the roots of both L1-2 and L2-6 lines (Fig. 4F). In either the roots or midribs of transgenic plants, the abundances of Burkholderiaceae and Micropepsaceae increased significantly (Fig. 4F). Populations of bacterial genera in transgenic plants were further investigated compared with WT controls. Unclassified Rhizobiaceae and *Streptomyces* had reduced populations in the roots of both L2-6 and L1-2 lines, while the populations of *Burkholderia*–*Caballeronia*–*Paraburkholderia* and *Castellaniella* belonging to Burkholderiales increased in the L2-6 line. Similar results were observed in unclassified Rhodanobacteraceae in the L2-6 and L1-2 lines (Fig. 4F, Supplementary Data Fig. S7, and Supplementary Data S7). Taken together, the results suggested that LasLYS1 and LasLYS2 had a selective effect on the microbial community of transgenic plants, which mainly diminished the population of Xanthomonadaceae (*Stenotrophomonas* and *Luteimonas*) and Rhizobiaceae while augmenting Burkholderiaceae and Rhodanobacteraceae in the microbial community of the root.

### Characteristics of defense-related transcription activities in transgenic plants

**Figure 5:**
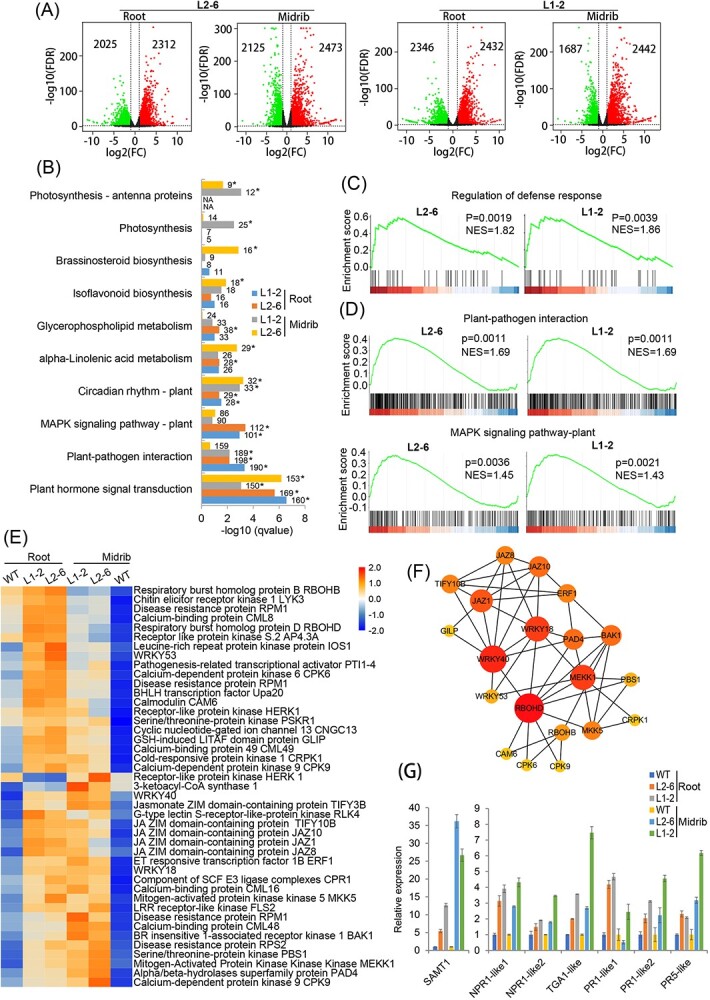
Characteristics of transcriptomic profiles affected by *LasLYS1* and *LasLYS2* in transgenic plants. (A) Volcano plots of gene expression in roots and midribs of L1-2 and L2-6 transgenic lines compared with WT control. Green and red plots represent down- and upregulated DEGs [|log_2_(fold change)| > 1 and FDR < 0.01], respectively. (B) Representative pathways regulated by *LasLYS1* and *LasLYS2*. All the DEGs from the roots and midribs of LI-2 and L2-6 transgenic lines were used for KEGG pathway enrichment analysis. *Significantly regulated pathways (*q*-value <0.05). (C) and (D) Gene set enrichment analyses (GSEA) of DEGs involved in regulation of defense response, plant–pathogen interaction and MAPK signaling processes in roots (C) and midribs (D) from L1-2 and L2-6 transgenic lines compared with WT control. NES, normalized enrichment score. (E) Hierarchical clustering of the core defense DEGs shared by the roots and midribs of LI-2 and L2-6 transgenic lines. These core DEGs were extracted based on the GSEA results from (C) and (D). Values are row-scaled to show relative expression. Blue and red indicate low and high levels, respectively. (F) Network diagram of overlapped defense DEGs. The DEGs from (E) were used to construct the network diagram based on *Arabidopsis* homologies on the STRING platform. Different colored bubbles indicate *K*-means clustering of the network (*n* = 3). Minimum required interaction score, ≥0.04. (G) Relative expression of seven key DEGs involved in salicylic acid-mediated defense response. Gene expression was determined by qRT–PCR. Relative expression in transgenic plants was estimated compared with WT controls using citrus *GAPDH* as the internal reference. Values are expressed as means ± standard deviation of three biological replicates.

Endophytic bacteria community changes in transgenic plants may influence the host immune response [27,28]. Thus, RNA-seq experiments were used to investigate the features of the defense response in the L1-2 and L2-6 lines. The RNA-seq study was performed on healthy roots and midribs in three biological replicates. Supplementary Data S8 provides a summary of the sequencing data in each duplicate. Principal component analysis revealed that gene expression in the L1-2 and L2-6 lines differed significantly from that in WT controls (Supplementary Data Fig. S8). Compared with WT controls, In the roots and midribs of the two transgenic plants, 4337, 4598, 4778, and 4129 differentially expressed genes (DEGs) were found in comparison with WT controls (Fig. 5A and Supplementary Data S9 and S10). Roots and midribs shared 1371 DEGs (Supplementary Data Fig. S9). The expression of 28 randomly chosen DEGs was examined by qRT–PCR to confirm the RNA-seq findings. The results demonstrated that the expression patterns of 26 DEGs matched those found in RNA-seq data (Supplementary Data Fig. S10).

*LasLYS1* and *LasLYS2* expressions were strongly correlated with ‘plant hormone signal transduction’, ‘plant–pathogen interaction’, and ‘MAPK signaling pathway–plant’ pathways, according to KEGG pathway enrichment analysis (Fig. 5B, Supplementary Data Fig.S8, and Supplementary Data S11). The expression of LasLYS1 and LasLYS2 substantially elevated the ‘regulation of defense response’ process in roots, as well as the ‘plant–pathogen interaction’ and ‘MAPK signaling pathway–plant’ processes in midribs, according to gene set enrichment analyses (GSAEs) (Fig. 5C and D). The root and midrib of LI-2 and L2-6 lines shared 43 core genes from the three functional pathways (Supplementary Data S12). All of these genes showed higher expression in both roots and midribs, with the exception of *HERK1*, which showed reduced expression in roots (Fig. 5E and Supplementary Data S12). Six of the core genes (*PTI1-4*, *WRKY18*, *WRKY40*, *WRKY53*, *Upa20*, and *ERF1*) encode transcription factors; four (three *RPM1s* and one *RPS2*) encode disease resistance proteins; two (*RBOHB and *RBOHD**) encode respiratory burst oxidase homologs; and 17 are kinase family protein genes (Fig. 5E). A network diagram comprising the 43 genes was also created using *Arabidopsis* homologies. The network analysis showed that the critical genes in the network were *WRKY40*, *WRKY18*, *MEKK1*, and *RBOHD* (Fig. 5F and Supplementary Data S13).

In addition, the RNA-seq data showed that several genes involved in salicylic acid (SA)-mediated defense responses were obviously affected by *LasLYS1* and *LasLYS2* expression (Supplementary Data S14). SA-mediated defense response played a vital role in the resistance of citrus to HLB [7,10,29]. Thus, we verified the expression of these genes using qRT–PCR. The data showed that the expressions of SAMT1, *NPR1-like1*, *NPR1-like2*, *TGA1-like*, *PR1-like1*, *PR1-like2* and *PR5-like* genes were dramatically upregulated in LasLYS1 and LasLYS2 transgenic plants (Figure 5G), revealing that LasLYS1 and LasLYS2 augment the SA-mediated defense response.

### Sequence characteristics of LasLYS1 and LasLYS2

**Figure 6:**
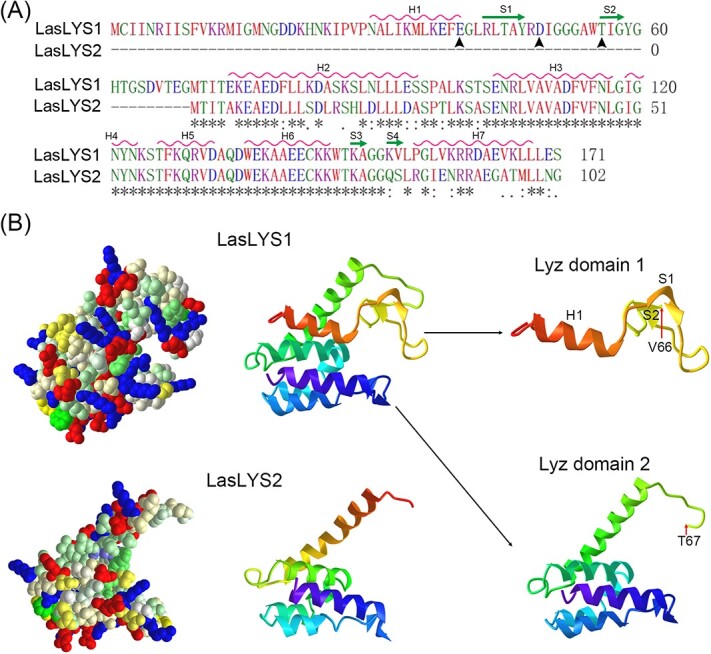
Analysis of LasLYS1 and LasLYS2 protein sequences. (A) Comparative analysis of the primary and secondary structures of LasLYS1 and LasLYS2. Arrows indicate catalytic residues. *, : and . indicate a completely conserved, high, and low similarity among residues, respectively. H# and S# indicate α-helix and β-sheet, respectively. (B) Predicted tertiary structures of LasLYS1 and LasLYS2. Lyz domains 1 and 2 indicate the tertiary domains of lyz-endolysin-autolysin.

The study presented above highlights significant differences in the bactericidal capabilities of the two endolysins. Comparative analysis revealed that LasLYS1 and LasLYS2 belong to the lysozyme group and share high residue similarity with T4 lysozyme from *Enterobacteria* phage T (Supplementary Data Fig. S11). However, examination of the *Ca*Las endolysin sequences did not reveal a conserved N-terminal signal-arrest-release domain responsible for endolysin export to the membrane and release into the periplasm [30] (Supplementary Data Fig. S11). The amino acid sequence of LasLYS2 shares 77.4% identity with the 3′ sequence (70–171 amino acids) of LasLYS1, with a 49-amino acid long identical sequence, SENRLVAVADFVFNLGIGNYNKSTFKQRVDAQDWEKAAEECKKWTKAGG (Fig. 6A). In comparison with LasLYS2, LasLYS1 possesses an additional N-terminal extension of 70 amino acid residues, of which three catalytic residues (Glu40, Asp49, and Thr56) were predicted (Fig. 6A). These catalytic residues were not found in LasLYS2. No signal peptide or transmembrane domain was identified in either protein. Secondary structure prediction indicated that LasLYS1 possesses seven α-helices (H1–H7) and four β-sheets (S1–S4), while LasLYS2 contains six α-helices (H2–H7) and two β-sheets (S3 and S4), lacking H1 α-helix and S1 and S2 β-sheets (Fig. 6A). Tertiary structure prediction revealed that LasLYS1 consists of two domains: lyz-endolysin-autolysin (Lyz) domains 1 and 2. These domains correspond to the sequences from 24 to 66 amino acids (including H1 α-helix and S1 and S2 β-sheet) and from 67 to 170 amino acids (including H2–H7 α-helices and S3 and S4 β-sheets), respectively (Fig. 6B). On the other hand, LasLYS2 only possesses Lyz domain 2 (Fig. 6B). This analysis suggests that Lyz domain 2 plays a significant role in the functionality of both LasLYS1 and LasLYS2.

### Lyz domain 2 is the major bactericidal motif of LasLYS1 and LasLYS2

**Figure 7:**
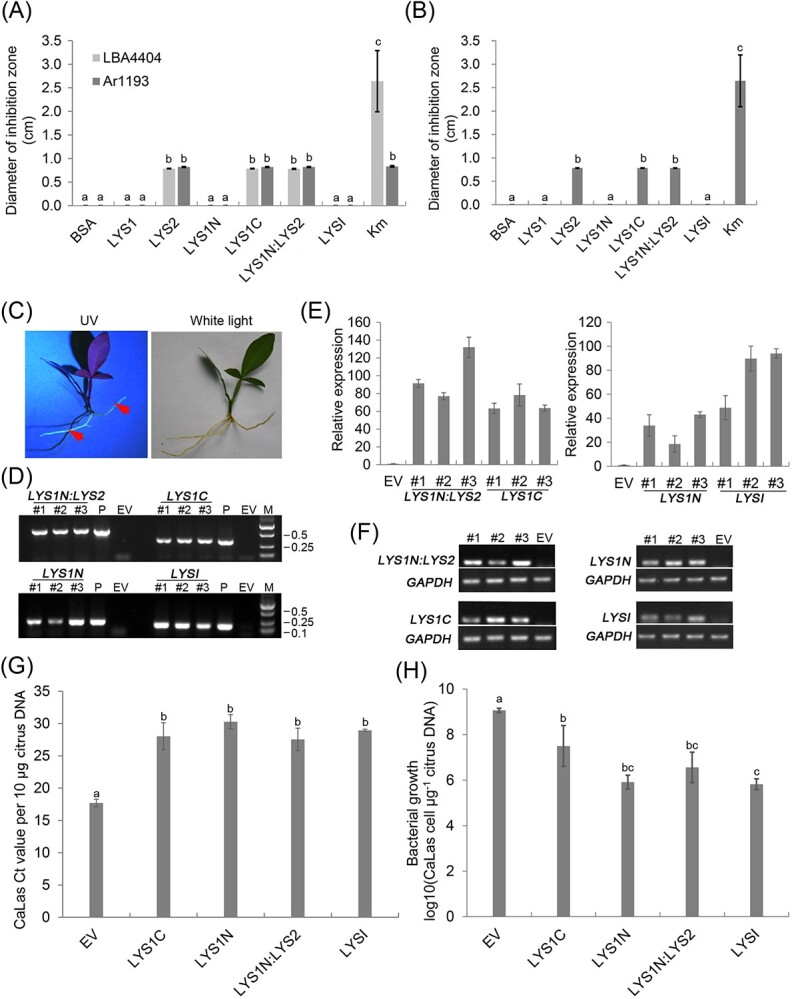
Analysis of bactericidal activities of different LasLYS variants. (A) and (B) Bactericidal activities of different LasLYS variants by the Oxford cup method. Strains LBA4404 (A) and Ar1193 (A) were tested, as well as the *Xcc* (B) strain. A 50-μl solution containing 1.5 mg/ml BSA, 1.5 mg/ml *LasLYS*, and 0.5 mg/ml kanamycin (3.0 mg/ml *LasLYS* for Xcc) was added to the Oxford cup. The size of the inhibition zone was measured after 3 days of inoculation. Values are expressed as means ± standard deviation of three independent tests. (C)–(H) Resistance evaluation of different LasLYS variants against *Ca*Las. To evaluate the resistance of *LasLYS* variants against CaLas, *R. rhizogenes*-mediated transformation was performed using *Ca*Las-infected Carrizo citrange stems as explants. Transgenic hair roots were screened for GFP fluorescence, with GFP-positive hairy roots indicated by red arrows (C). The presence of *LasLYS* genes in GFP-positive roots was confirmed by PCR. Three groups (#1, #2, and #3), each consisting of three to five independent transgenic roots, were examined (D). The expression levels of *LasLYS* genes in transgenic roots were analyzed by qRT–PCR (E) and RT–PCR (F), with citrus *GAPDH* used as the internal reference for transcript normalization. The *Ca*Las content in transgenic hair roots was determined using qPCR, with the CT value of the *Las16S* gene (G) and Las cells μg^−1^ of citrus DNA (H) used as indicators. Values are expressed as the means ± standard deviation of the three groups (#1, #2, and #3). Different letters at the top of the bars indicate significant differences from the EV control, as determined by Duncan’s test (*P* < .05). LYS1, LasLYS1; LYS2, LasLYS2; LYS1N, LasLYS1N; LYS1C, LasLYS1C; LYSI, LasLYSI; LYS1N:LYS2, fusion of LasLYS1N, and LasLYS2; EV, transgenic roots containing pNMG empty vector as a control.

To further understand the role of Lyz domains 1 and 2 in the function of LasLYS1 and LasLYS2 (Fig. 6), we constructed the *LasLYS1N*, *LasLYS1C*, and *LasLYSI* genes, which encode amino acids 1–66 and 67–171 in LasLYS1 and the 49-amino acid identical sequence shared by LasLYS1 and LasLYS2, respectively (Supplementary Data Fig. S12). The LasLYS1N and LasLYS1C proteins contain Lyz domains 1 and 2, respectively (Fig. 6). *LasLYS1N* was also fused into the 5′-terminal of *LasLYS2* to generate the *LasLYS1N:LYS2* hybrid gene (Supplementary Data Fig. S12) to evaluate the effect of Lyz domain 1 on LasLYS2 function. Their encoded proteins were produced through the *E. coli* expression system. Their bactericidal activities against *LBA4404*, *Ar1193*, and *Xcc* were determined by the Oxford cup method (Fig. 7A and B and Supplementary Data Fig. S12). LasLYS1C had bactericidal activity against *LBA4404*, *Ar1193*, and *Xcc* that was comparable to that of LasLYS2. But no visible bactericidal activities were detected in LasLYS1, LasLYS1N, or LasLYSI. The results revealed that the Lyz domain 2 of LasLYS1C had bactericidal activity, but Lyz domain 1 may repress the function of Lyz domain 2 in LasLYS1. However, the LasLYS1N:LYS2 fused protein displayed bactericidal activity against *LBA4404*, *Ar1193*, and *Xcc*, which was also comparable to that of LasLYS2, indicating that Lyz domain 1 had no inhibitory effect on Lyz domain 2 of LasLYS2. Overall, our data showed that Lyz domain 2 is the key bactericidal motif of the endolysins.

To determine the bactericidal activities of the Lyz domains 1 and 2 against *Ca*Las, *LasLYS1N*, *LasLYS1C*, *LasLYSI*, and *LasLYS1N:LYS2* driven by the 35S promoter were introduced into *Ca*Las-infected Carrizo citrange using a *Rhizobium rhizogenes*-mediated transformation [31]. The pNMG empty vector (EV) was also transformed into *Ca*Las-infected Carrizo citrange as the control. Two months after transformation hairy roots 2–4 cm long were generated from the ends of explants (Fig. 7C), and transgenic hair roots were identified by observing GFP fluorescence since the vectors have a GFP reporter gene (Fig. 7C and Supplementary Data Fig. S13). The integration and expression of *LasLYS* genes in hairy roots were identified by PCR (Fig. 7D–F). Ten to fifteen independent transgenic hairy roots per *LasLYS* construct were obtained in this test. Quantitative analysis showed that *Ca*Las contents had no significant difference among the midribs from *Ca*Las-infected explants generating transgenic hairy roots, including EV controls (Supplementary Data Fig. S13). But in *LasLYS1N*, *LasLYS1C*, *LasLYSI*, and *LasLYS1N:LYS2* transgenic hairy roots, *Ca*Las contents were significantly lower than those in EV controls (Fig. 7G and H), indicating that both Lyz domain 1 and domain 2 have bactericidal activities against *Ca*Las in citrus. The data also revealed that LasLYSI and LasLYS1N had high bactericidal activity against *Ca*Las.

## Discussion

Bactericidal peptides or proteins from other species have displayed great potential in citrus breeding for broad-spectrum disease resistance. Our previous study showed that expressing synthesized cecropin B genes in transgenic citrus alleviated the susceptibility to HLB and citrus canker [32, 33]. Hao *et al.* [34] showed that overexpression of a modified citrus thionin enhanced the resistance to both citrus canker and HLB diseases in transgenic Carrizo citranges. The above-mentioned antimicrobial peptides kill bacteria by disrupting the integrity of the bacterial membrane [35]. Endolysin, which kills bacteria by lysing the cell wall, provides another strategy for improving plant disease resistance [19, 36]. Especially, Wittmann *et al.* [19] demonstrated that the CMP1 endolysin gene confers resistance to its own host *C. michiganensis* subsp*. michiganensis* in transgenic tomatoes. This inspired us to assess the roles of the endolysin genes *LasLYS1* and *LasLYS2* derived from *Ca*Las in developing resistance to HLB in citrus. Our study showed that the expression of *LasLYS1* and *LasLYS2* in Carrizo citrange led to resistance to HLB, though *LasLYS2* transgenic plants had higher resistance. Inhibition zone tests revealed that LasLYS1 and LasLYS2 had selective bactericidal activity against some Rhizobiaceae bacteria, which was further confirmed by endobacterial diversity analysis of transgenic plants. *Ca*Las is an early branch of the family Rhizobiaceae [24]. These data, therefore, indicated that LasLYS1 and LasLYS2 have bactericidal activity against *Ca*Las. Surprisingly, we found that LasLYS2 at high concentrations demonstrated bactericidal activity against *Xcc*. Thus, we further evaluated the resistance of transgenic plants to citrus canker. The results showed that *LasLYS2* transgenic plants were resistant to citrus canker, but *LasLYS1* transgenic plants only exhibited weak resistance. Indeed, three LasLYS2 transgenic plants, L2-1, L2-3, and L2-6, simultaneously displayed high resistance to HLB and citrus canker. Thus, our study confirmed that LasLYS2 has dual resistance to HLB and citrus canker. These transgenic lines with disease resistance observed under controlled conditions are currently being evaluated in field trials. Furthermore, based on the structures of LasLYS1 and LasLYS2, we synthesized four endolysins (LasLYS1N, LasLYS1C, LasLYSI, and LasLYS1N:LYS2) and confirmed that they also possess potential resistance to HLB and citrus canker, which provided new candidates for genetic improvement of disease resistance in citrus.

The function prediction showed that LasLYS1 and LasLYS2 are lysozymes. It has been shown that the lysozyme genes, which originated from different species, such as bacteriophage, chicken, goose, and human, confer broad resistance against different pathogenic bacteria and even fungi [19, 36–38]. The reported results, including the present study, indicate that lysozyme genes are an elite candidate for developing disease resistance in citrus. To enhance the bactericidal efficacy of lysozyme in transgenic plants, the subcellular localization of lysozyme is selected based on the colonization sites of the targeted pathogen in the plant host [36]. Lysozyme genes are usually fused to a signal peptide encoding sequence to accumulate their proteins in intercellular space, where the pathogen colonizes [36–38]. For *Ca*Las, which multiplies in the cytoplasm of sieve elements in the phloem once it enters the plant [1], lysozyme should be retained in the citrus cytoplasm to effectively battle bacteria. Thus, to retain LasLYS1 and LasLYS2 in the citrus cytoplasm, no signal peptide was used in this study. However, as a result, LasLYS2 transgenic plants still had resistance to *Xcc*, which multiplies in the intercellular space of citrus, indicating that LasLYS2 can kill *Xcc* without the help of a signal peptide. It is possible that LasLYS2 is transported outside the host cell to execute its bactericidal function through passive diffusion due to the characteristics of its primary and tertiary structures. In fact, it would be worthwhile to increase expression of endolysin genes without being concerned about the transport of the enzyme by adding an effective signal peptide for efficiently lysing bacteria at the site of infection [19]. Thus, our data demonstrated an expression strategy for the utilization of endolysins to simultaneously improve resistance to various pathogens with different life habits, such as *Ca*Las and *Xcc*, which live in the cytoplasm and intercellular space of host cells, respectively.

LasLYS2 displayed a wider bactericidal spectrum and higher resistance to HLB and citrus canker, despite having similar secondary and tertiary structures to LasLYS1. Informatics analysis revealed that LasLYS2 only possesses Lyz domain 2, while LasLYS1 has both Lyz domains 1 and 2. Bacteriostatic zone tests demonstrated that Lyz domain 2 was crucial for the activity of both LasLYS1 and LasLYS2. Lyz domain 2 consists of five α-helices (H2–H7 α-helix), whereas Lyz domain 1 only contains an H1 α-helix. This suggests that the H2–H7 α-helix plays an important role in the function of Lyz domain 2. Furthermore, LasLYSI, lacking the H2 α-helix, lost its activity against *LBA4404*, *Ar1193*, and *Xcc*, although it still inhibited *Ca*Las growth in citrus. This further suggests that the H2 α-helix could be a core structure essential for the function of Lyz domain 2. Further studies are needed to confirm the role of the α-helices in endolysin activity. Lyz domain 1 in LasLYS1 contains the predicted catalytic triad Glu-8aa-Asp/Cys-6aa-Thr, responsible for the hydrolysis reaction [39]. However, bacteriostatic zone analysis revealed that Lyz domain 1 exhibited no *in vitro* activity against the tested strains in LasLYS1N and LasLYS1N:LYS2. Lyz domain 1 also showed potential inhibition of Lyz domain 2 in LasLYS1 but not in LasLYS2, explaining the functional difference between LasLYS1 and LasLYS2. The different impacts of Lyz domain 1 on the activity of Lyz domain 2 in LasLYS1 and LasLYS2 may be related to the distinct amino acid sequences in LasLYS1 and LasLYS2 (Fig. 6A), which will be investigated in future experiments. Additionally, the exogenous application of endolysins against Gram-negative bacteria is challenging due to the outer membrane acting as a barrier that prevents endolysins from accessing the peptidoglycan [12]. The smaller size of LasLYS2 (11.4 kDa) compared with LasLYS1 (19.1 kDa) (Fig. 6A) suggests that LasLYS2 may have better permeability through the bacterial outer membrane, enabling it to more effectively kill cells. Similarly, when endolysins are expressed by transgenic plants, they must overcome the barrier of the bacterial outer membrane to exert their antibacterial effects. Therefore, in theory, the smaller size of LasLYS2 should make it more competent at killing *Ca*Las and *Xcc* in citrus cells. This presumption is also supported by the *A. rhizogenes*-mediated transformation experiment, which showed that the smaller proteins, LasLYS1N (7.7 kDa) and LasLYSI (5.7 kDa), exhibited stronger inhibitory activity against *Ca*Las compared with the larger proteins, LasLYS1C (11.5 kDa) and LasLYS1N:LYS2 (19.0 kDa). Furthermore, we speculate that Lyz domain 2 contains unidentified catalytic residues, as LasLYS2, LasLYS1C, and LasLYSI all demonstrated bacteriolytic activity. The catalytic triad of lysozyme typically involves Glu as a general acid responsible for the catalytic mechanism, while Asp is not essential [40]. Therefore, it remains to be determined in future studies which of Glu and Thr residues in Lyz domain 2 is essential for its catalytic function. For instance, residues Glu35 (Glu2 in LasLYSI), Asp43 (Asp10 in LasLYSI), and Thr57 (Thr24 in LasLYSI) in Lyz domain 2 potentially form a catalytic triad [41]. In conclusion, our data suggest that the structure of Lyz domain 2 is more efficient for genetically improving broad-spectrum tolerance or resistance to HLB and canker in citrus.

Plant endophytes are activists of host health in a commensal or beneficial manner, which can inhibit pathogen infection through direct competition, secreting effectors or antimicrobial compounds, or stimulating plant immunity [42, 43]. As antimicrobial proteins, endolysins may play similar roles. Here, to characterize the endophytic bacterial community affected by expression of *LasLYS1* and *LasLYS2* in citrus transgenic plants, we performed 16S amplicon sequencing of the midrib and root since the tissues are the main habitat of *Ca*Las [1] and the root is preferentially colonized by *Ca*Las [25, 26]. The analysis showed that LasLYS1 and LasLYS2 significantly altered the endophytic bacterial community of roots but had little effect on that of midribs. Our data demonstrated that LasLYS1 and LasLYS2 repressed Xanthomonadaceae and Rhizobiaceae bacteria. Moreover, abundances of populations of Burkholderiaceae and Rhodanobacteraceae increased in both the roots and midribs of *LasLYS1* and *LasLYS2* transgenic plants. Members of the Burkholderiaceae family are believed to be beneficial bacteria for defending against HLB disease [27, 44, 45]. For example, *Burkholderia territorii* strain A63 and *Burkholderia metallica* strain A5 have antagonistic activities against *Sinorhizobium meliloti*, a relative of *Ca*Las [27], and significantly suppress citrus canker in leaves challenged with *Xcc* [46]. Rhodanobacteraceae were detected in the root communities of asymptomatic trees, but absent in diseased trees after *Ca*Las infection [45]. These results suggested that the increase in Burkholderiaceae and Rhodanobacteraceae families might be favorable for resistance to HLB in citrus.

External application of host-derived antimicrobial peptides or proteins can prime citrus innate immunity to reduce *Ca*Las infection [28,47]. The present study demonstrated that expression of *LasLYS1* and *LasLYS2* augmented defense-related transcription activities in healthy transgenic plants. Inoculation of Burkholderiaceae strains can activate the expression of genes involved in the induced systemic resistance of citrus, which has the potential to promote plant health [27, 46]. Several key genes (such as *SAMT1*, *NPR1*, *TGA1*, *PR1*, and *PR5*), participating in SA-dependent systemic/induced acquired resistance, were upregulated in our transgenic plants. Therefore, increasing some beneficial bacteria, such as Burkholderiaceae and Rhodanobacteraceae, might promote an SA-mediated defense response in transgenic plants. We also demonstrated that the MAPK signaling pathway was upregulated by *LasLYS1* and *LasLYS2* expression in transgenic plants. The activation of the MAPK signaling pathway plays a positive role in citrus resistance to HLB [48]. Further, the results of this study suggested that WRKY40, WRKY18, MEKK1, and RBOHD have crucial roles in MAPK signaling in transgenic plants. During *Ca*Las infection, expressions of MAPKK19 (an MEKK), WRKY40, and RBOHD homologs increased in rough lemon, which is HLB-tolerant, compared with that of ‘Madam Vinous’ sweet orange, which is HLB-susceptible [48]. Expression of *RBOHD* induced by *Ca*Las infection is possibly responsible for ROS accumulation in citrus phloem [49]. ROS accumulation causes cell death in phloem tissue, which is a typical symptom of HLB disease [49]. Constitutive expression of WRKY18 in *Arabidopsis* enhanced resistance to *Pseudomonas syringae* [50,51]. Thus, it is meaningful to verify the functions of these genes involved in citrus resistance to HLB in future studies.

### Conclusions

This study demonstrated that transgenic expression of LasLYS2 endolysin from *Ca*Las can simultaneously provide good protection for citrus plants against HLB and citrus canker that are induced by *Ca*Las and *X. citri* pv. *citri*, respectively. Importantly, the *LasLYS2* transgene can maintain low *Ca*Las titers in citrus plants without visible symptoms for a long time, and it is even possible to completely clear *Ca*Las from infected plants. The study also demonstrated that *LasLYS1* and *LasLYS2* positively affected the host’s endophytic bacteria community and defense transcription activities, which were beneficial to plant resistance. We highlighted the observed characteristics of Lyz domain 2 of the *Ca*Las endolysins that are associated with its structure and function, which will provide a good reference for screening existing endolysins or bactericidal proteins for genetically generating disease resistance in citrus.

## Materials and methods

### Plant and bacterial materials

In the National Citrus Variety Improvement Center’s nursery in Chongqing, China, Carrizo oranges were grown for citrus transformation. Sweet orange scions infected with *Ca*Laswere taken from a citrus plantation in China’s Guangxi Province. Grafting the scions onto Jincheng orange (*Citrus sinensis*) seedlings in a greenhouse was done to preserve and reproduce *Ca*Las. This research employed the *Xcc* strain XccYN1 [9].

### Cloning and analysis of *LasLYS1* and *LasLYS2*

Based on the coding sequences of *LasLYS1* (CLIBASIA_04790) and *LasLYS2* (CLIBASIA_04800), the two genes were amplified from *Ca*Las-infected Wanjincheng orange midribs by PCR, cloned into pGEM-T^®^ Easy Vector (Promega, WI, USA), and confirmed by Sanger sequencing. The TMHMM server (v.2.0) and signalP5.0 tool were used to predict the transmembrane structure and signal peptide of LasLYS1 and LasLYS2, respectively. The Phyre5 tool [52] was used to predict the secondary and tertiary structures of LasLYS1 and LasLYS2. Multiple sequence alignments of proteins were performed by the BLAST program. Based on the neighbor-joining method, the phylogenetic tree of proteins was constructed using MEGA7.0 software [53].

### Protein expression and bactericidal activity analysis

Based on the sequences of *LasLYS1* and *LasLYS2*, we synthesized the *LasLYS1N*, *LasLYS1C*, *LasLYSI*, and *LasLYS1N:LYS2* genes by PCR. These genes, including *LasLYS1* and *LasLYS2,* were cloned into the pCZN1 vector. The resulting constructs were transferred into the Arctic-Express (DE3) strain. Protein expression was induced by 0.4 mM isopropyl-thio-2-d-galactopyranoside for 4 h at 37°C. Recombinant proteins with a His-tag were purified using Ni^2+^-NTA agarose following the manufacturer’s instructions (Novagen, Madison, WI, USA). Protein purity and concentrations were determined by SDS–PAGE and the Bradford assay [54], respectively.

Recombinant proteins’ bactericidal properties were evaluated using the Oxford cup technique. In a shaking incubator at 28°C for 16 h, bacterial strains were grown in Luria–Bertani broth (LB, Oxoid, Basingstoke, UK) medium. Centrifugation was used to separate the bacteria for 1 min at 10 000 rpm, after which they were diluted in LB to an OD_600_ of 0.2. Five Oxford cups were placed onto 25 ml of LB solid medium and 2 ml of bacterial resuspension to create LB plates. The Oxford cup received separate additions of 50 μl recombinant protein. The inoculation was kept at 28°C for 3 days before being photographed. Using a Vernier caliper, the inhibitory zone’s diameter was determined. The test was repeated three times.

### Citrus transformation

The sequences of *LasLYS1*, *LasLYS2, LasLYS1N*, *LasLYS1C*, *LasLYSI*, and *LasLYS1N:LYS2* were optimized based on codon usage bias in plants, and the optimized sequences were artificially synthesized by PCR. The optimized *LasLYS1* and *LasLYS2* were cloned into a pLGN vector [32] to generate pLasLYS1 and pLasLYS2 constructs in which *LasLYS1* and *LasLYS2* transcription was driven by a strong 35S constitutive promoter. These constructs were transformed into the *A. tumefasciens* EHA105 strain for producing transgenic plants. The optimized *LasLYS1N*, *LasLYS1C*, *LasLYSI*, and *LasLYS1N:LYS2* were inserted into a pNMG vector, and their expressions were controlled by the 35S promoter (Supplementary Data Fig. S13). These expression vectors, including pNMG empty vector, were transformed into the *R. rhizogenes* K599 strain for regenerating transgenic hair roots.

With a few minor adjustments (Supplementary Data Fig. S3), pLasLYS1 and pLasLYS2 constructs were introduced into the citrus genome using epicotyl segments from Carrizo citrange seedlings as explants [32]. After resistance-selective culture, transgenic shoots were identified by GUS histochemical staining and then micrografted onto Wanjincheng orange seedlings and recovered in an MS liquid medium [55]. The stems of transgenic plants were then cut into five or six pieces, each with one or two leaves, when more than seven leaves had been produced. These stem cuttings were then put on rooting media [56]. After 2 months, rooted plantlets were moved to pots filled with nutrient soil in a greenhouse.

*A. rhizogenes*-mediated Carrizo citrange transformation was performed as described [31]. In brief, 2-cm-long branches with one or two leaves were removed from Carrizo citrange plants that had been infected with *Ca*Las for 2 years, and the cut ends of the branches were vacuum-soaked for 30 min at 30 psi with K599 (OD_600_ = 0.5) solution. The infected explants were put vertically into vermiculite in seedling-raising discs and then maintained in an artificial climate chamber with 80% relative humidity and a 16 h photoperiod at 26°C. Two months later, transgenic hairy roots were detected using GFP fluorescence detection and PCR analysis.

### Determination of resistance to huanglongbing in transgenic plants

Using the leaf-disc grafting method [25], three or four 1-year-old healthy plants per independent transgenic line (including healthy WT control) with comparable height and crown were inoculated with *Ca*Las-infected Wanjingcheng orange leaf discs. Prior to grafting, PCR was used to detect the presence of CaLas in the petioles of the leaves (Supplementary Data Fig. S14). Then, leaf discs were extracted from the *Ca*Las-containing leaves and grafted onto the leaves of transgenic plants as described [25]. Each plant was grafted with six leaf discs from various CaLas-containing leaves in order to minimize the influence of CaLas’ unequal distribution on the evaluation. To isolate DNA, three randomly selected roots or midribs from each plant were combined into a single sample at regular intervals. qPCR was used to determine the *Ca*Las *16S* and citrus *18S* contents of DNA samples, as reported [32]. Using WT plants as controls, the disease intensity for each independent line was estimated based on *Ca*Las populations from three or four plants. For the detection of *Ca*Las in hairy roots, DNA was isolated from three to five independent transgenic roots as a biological repeat. For each gene, three biological repeats were designed. The resistance level per gene was estimated using *Ca*Las populations from biological repeats in comparison with empty controls.

### Determination of resistance to citrus canker in transgenic plants

Using WT plants as a control, resistance to citrus canker in transgenic plants was evaluated by the pinprick method described in previous work [9]. Three fully mature, healthy leaves per transgenic line were tested. Twenty-four puncture sites were made for every leaf. One microliter of 1 × 10^8^ CFU/mL of XccYN1 suspension was added to each puncture site. Symptoms were recorded by photographing at 9 dpi, and the diseased area was measured using ImageJ 2.0 software (National Institutes of Health, MD, USA). Seventy-two punctures per independent transgenic line were sorted based on the diseased area using the following index: 0, <0.2 mm^2^; 1, 0.2–0.4 mm^2^; 2, 0.4–0.6 mm^2^; 3, 0.6–0.8 mm^2^; 4, 0.8–1.0 mm^2^; 5, 1.0–1.2 mm^2^; 6, 1.2–1.4 mm^2^; 7, >1.4 mm^2^. The disease index (DI) for each independent transgenic was calculated with the formula:

DI = Σ (no. of each index × the corresponding index)/(72 × 7) × 100.

The growth of XccYN1 in the leaves of transgenic plants was also evaluated daily (0–9 dpi) after infection, as described previously [9].

*In vivo* infiltration was also used to determine the canker resistance of transgenic plants [9]. XccYN1 suspensions (1 × 10^8^ CFU/ml) were injected into 3-month-old leaves. Daily observations of the development of canker symptoms in inoculated plants were made.

All of the preceding experiments were repeated three times.

### Microbiome analysis

Healthy transgenic and healthy WT plants were used to analyze the endophytic bacterial community of the roots and midribs. Three biological replicates were carried out. For each biological replicate, citrus DNA was extracted from at least three plants. The V3 and V4 regions of the bacterial 16S DNA were amplified with the primers 335F/769R (Supplementary Data S15) to construct sequence libraries, and then the libraries were paired-end (250 bp) sequenced using the Illumina Novaseq 6000 system (Illumina, Santiago, CA, USA) at Biomarker Technologies Co., Ltd (Beijing, China).

The processing and assembly of raw data were performed as described [27]. ASVs were generated using the DADA2 method [57]. The taxonomy of the ASVs was assigned based on the SILVA database [58] using the RDP classifier [59]. The relative abundance of each taxon across samples was determined using the TSS method [60] based on read count data. The α-diversity and β-diversity of taxa were calculated using the Shannon index and Bray–Curtis dissimilarity, respectively, with the phyloseq package (v.1.22.3). Differential abundance analysis was performed using the metagenomeseq tool from the BMKCloud platform (www.biocloud.net). ASVs with an adjusted *P* < .05 and bacterial taxa with *P* < .05 were considered to have a significant difference compared with the WT controls.

### RNA-seq analysis

The investigation was conducted using three biological replicates. BioMarker Technologies Illumina, Inc. (Beijing, China) was entrusted with the RNA-seq analysis of roots and midribs from healthy transgenic and WT control plants. Using HISAT 2.0.5 [61], all the pristine data were mapped to the *C. sinensis* genome (v.3.0) (http://citrus.hzau.edu.cn). The databases GO, KO, KOG/COG, Nt, Nr, Pfam, and Swiss-Prot were utilized to predict gene function. featureCounts [62] was used to quantify the number of reads per gene. Using the DESeq2 package [63], DEGs in transgenic plants were identified in comparison with WT controls. The DEGs were designated by |fold change ≥ 1| and an amended *P*-value <.01. On the BMKCloud platform (www.biocloud.com), principal component analysis of gene expression and KEGG pathway enrichment, GSEA, and Venn diagram and heat map construction of DEGs were conducted. Using the STRING platform (v.11) [64], protein–protein interaction (PPI) networks of DEGs were constructed based on *Arabidopsis* homologies.

### RT–qPCR analysis

The extraction of citrus RNA, synthesis of cDNA, and quantification of gene expression were carried out in accordance with previous methods [32]. The *GAPDH* gene [65] was used to normalize gene expression as an internal reference. The relative expression of transgenic plants relative to WT controls was calculated using the Ct method (2^−ΔΔCt^) [66]. Supplementary Data S15 contains a list of all primers used in the assay. The experiment was repeated three times.

## Supplementary Material

Web_Material_uhad159

## Data Availability

16S rDNA amplicon and RNA-seq raw data were deposited in the NCBI Bioproject database under the accession numbers PRJNA922109 and PRJNA922327, respectively.
