# Supplementary material for: An endolysin gene from Candidatus Liberibacter asiaticus confers dual resistance to huanglongbing and citrus canker
Source: Hortic Res. 2023 Aug 8;10(9):uhad159. doi: 10.1093/hr/uhad159 (PMC10500150; doi:10.1093/hr/uhad159)
Supplement: Web_Material_uhad159 [file web_material_uhad159.zip › Supplementary Tables and Figures.docx]

An endolysin gene from *Candidatus* Liberibacter asiaticus confers dual resistance to Huanglongbing and citrus canker

Lanzhen Xu^1, *^, Kaiqing Mo^1, *^, Danlu Ran^1^, Juanjuan Ma^1^, Lehuan Zhang^1^, Yijia Sun^1^, Qin Long^1^, Guojin Jiang^1^, Xiaochun Zhao^1,^ ^†^, Xiuping Zou^1,^ ^†^

^1^Citrus Research Institute, Southwest University/National Citrus Engineering Research Center, Chongqing, People’s Republic of China

*Lanzhen Xu and Kaiqing Mo contributed equally to this work.

^†^**Corresponding authors:** Xiaochun Zhao and Xiuping Zou (Tel: 18996337618)

Postal address: No. 15, Citrus Village, Xiema Street, Beibei District, Chongqing 400712, China

Table S1. Ct value of Las16S gene in the roots of transgenic lines by qPCR

| Line | 1 MAI | 3 MAI | 6 MAI | 12 MAI | 20 MAI | 25 MAI | 27 MAI | 29 MAI |
| --- | --- | --- | --- | --- | --- | --- | --- | --- |
| WT | 26.08±3.23^c^ | 21.43±1.38^c^ | 23.82±1.52^c^ | 24.33±2.14^e^ | 23.41±1.19 ^c^ | 22.44±1.80^b^ | 20.12±1.83^c^ | 18.66±2.15^c^ |
| L1-1 | 30.39±1.20^b^ | 27.30±3.72^b^ | 26.52±1.53^c^ | 26.36±2.46^de^ | 27.20±2.73^b^ | 23.44±1.21^b^ | 22.73±0.41^b^ | 20.70±0.57^c^ |
| L1-2 | 33.28±2.33^ab^ | 33.36±1.41^a^ | 29.46±0.59^b^ | 28.93±1.96^cd^ | 26.98±1.98^b^ | 24.41±1.09^b^ | 23.24±0.31^b^ | 20.92±0.58^c^ |
| L1-4 | 34.19±1.00^a^ | 27.67±1.77^b^ | 25.65±1.54^c^ | 24.43±1.31^e^ | 26.78±1.07^b^ | 24.93±1.03^b^ | 25.78±0.61^a^ | 23.65±1.74^b^ |
| L2-1 | 32.10±0.32^ab^ | 32.76±0.29^a^ | 32.46±0.56^a^ | 33.41±0.69^ab^ | 32.16±1.98^a^ | 29.59±2.13^a^ | 28.99±0.88^a^ | 27.72±1.16^a^ |
| L2-3 | 32.23±0.61^ab^ | 33.16±0.70^a^ | 32.21±1.85^a^ | 31.07±1.50^bc^ | 31.66±1.16^a^ | N.D. | N.D. | N.D. |
| L2-6 | 35.26±1.21^a^ | 32.69±2.40^a^ | 34.24±2.51^a^ | 36.30±0.75^a^ | 34.42±1.51^a^ | N.D. | N.D. | N.D. |

Table S2. Ct value of Las16S gene in the midribs of transgenic lines by qPCR

| Line | 25 MAI | 27 MAI | 29 MAI |
| --- | --- | --- | --- |
| WT | 20.36±0.38^c^ | 21.55±0.35^c^ | 20.92±0.87^c^ |
| L1-1 | 21.94±0.87^b^ | 23.92±0.38^b^ | 21.99±0.38^c^ |
| L1-2 | 21.54±1.15^bc^ | 25.62±1.61^b^ | 23.42±0.16^c^ |
| L1-4 | 22.76±0.62^b^ | 28.08±1.29^a^ | 27.42±1.63^b^ |
| L2-1 | 26.24±0.23^a^ | 30.85±0.40^a^ | 30.34±2.07^a^ |
| L2-3 | N.D. | N.D. | N.D. |
| L2-6 | N.D. | N.D. | N.D. |

In Table S1 and S2, the mean threshold cycle values (Ct) of Las16S gene were determined by qPCR. Values are expressed as means ± standard deviation of three or four plants per line. Different letters to the upper-right of the values indicate significant differences from the wildtype (WT) control based on a Duncan’s test (P < 0.05). MAI indicates month after infection. N.D. indicates that CaLas bacteria were not detected.


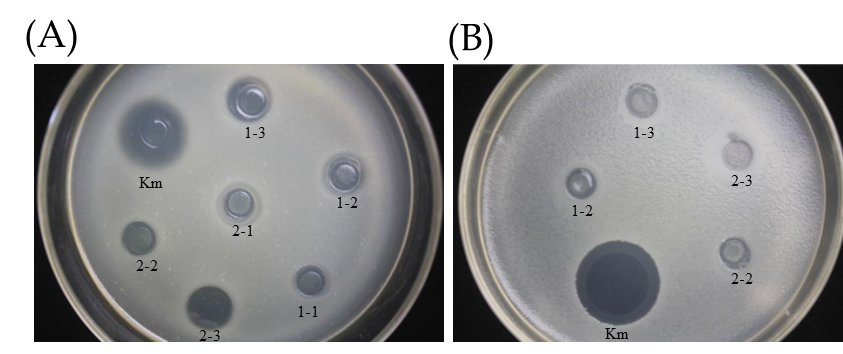


**Figure S1** Antibacterial activity analysis of LasLYS1 and LasLYS2 against the *Agrobacterium* EHA105 **(A)** and *Bacillus thuringiensis* HD73 strains **(B)**. Using kanamycin (Km) as positive control, the antibacterial activity of LasLYS1 and LasLYS2 were determined using the Oxford cup method. Here, the presented inhibition zone was photographed after three days of inoculation. Km: 0.5 mg/ml kanamycin; 1-1, 1-2 and 1-3 had 0.5, 1.0 and 1.5 mg/ml LasLYS1, respectively; 2-1, 2-2 and 2-3 had 0.5, 1.0 and 1.5 mg/ml LasLYS2, respectively. The test was repeated three times.


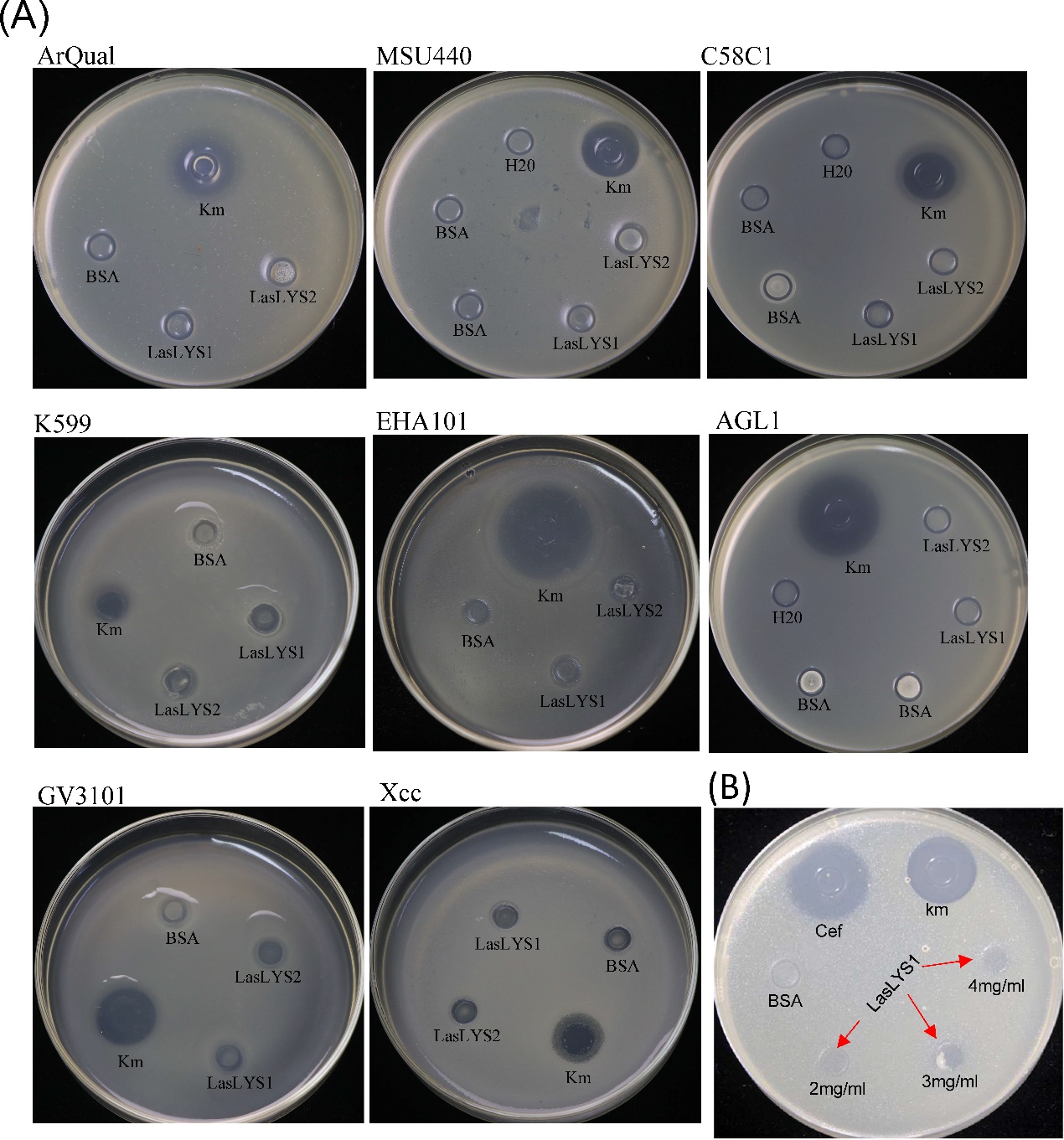


**Figure S2** Antibacterial activity analysis of LasLYS1 and LasLYS2 against eight bacteria using the Oxford cup method. **(A)** The antibacterial activity of 1.5 mg/ml LasLYS1 and LasLYS2 against ArQual, MSU4404, K599, C58C1, GV3101, AGL1 and EHA101 strains. **(B)** The antibacterial activity of different concentrations of LasLYS1 against *Xanthomonas citri* subsp. *Citri* (*Xcc*). Using 0.5 mg/ml kanamycin (Km) as the positive control and 1.5 mg/ml Bovine Serum Albumin (BSA) or H_2_O as the negative control, the presented inhibition zone was photographed after three days of inoculation. *Agrobacterium rhizogenes* Ar1193, ArQual, MSU4404, K599 and C58C1, *Agrobacterium tumefaciens* LBA4404, GV3101, AGL1 and EHA101 and *Xcc* strains were tested here. The test was repeated three times.


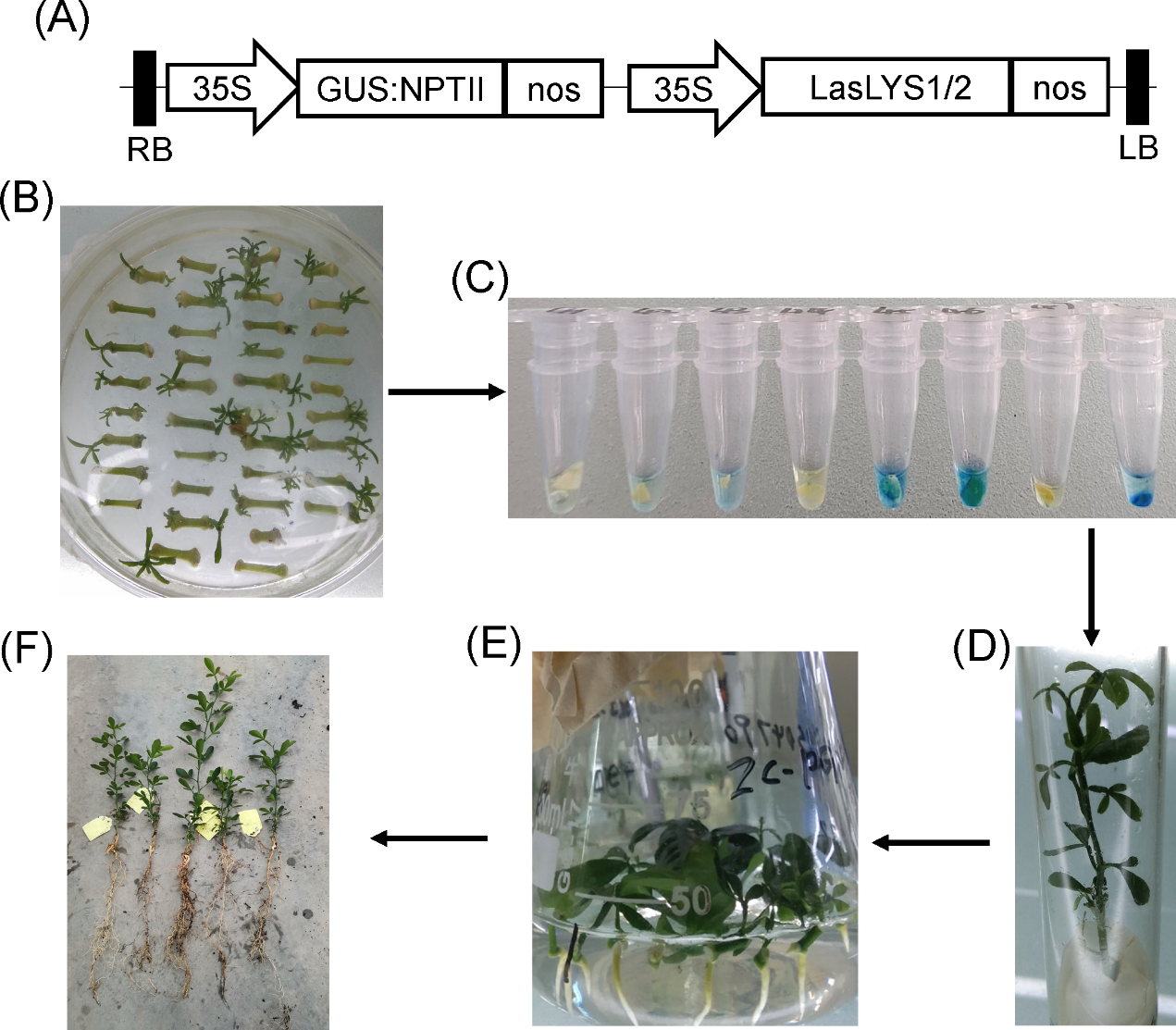


**Figure S3** Production of transgenic plants from Carrizo citrange epicotyls. **(A)** T-DNA structure of plant expression vectors containing *LasLYS1* and *LasLYS2*. 35S, CaMV 35S promoter; GUS:NPTII, the fusion of β-glucuronidase and neomycin phosphotransferase genes (for the screening of citrus transformants); NOS, the nopaline synthase terminator; LB, left border; RB, right border. **(B)** Regeneration of shoots in selection medium containing 50 mg/L kanamycin; **(C)** GUS histochemical staining of transgenic shoots; **(D)** micrografting of transgenic shoots on the top of Wanjincheng orange (*C. sinensis* Osbeck) seedlings *in vitro*; **(E)** rooting of branch sections from the transgenic shoots in **(D)** on root medium; **(F)** rooted transgenic plants grown for six months in greenhouse.


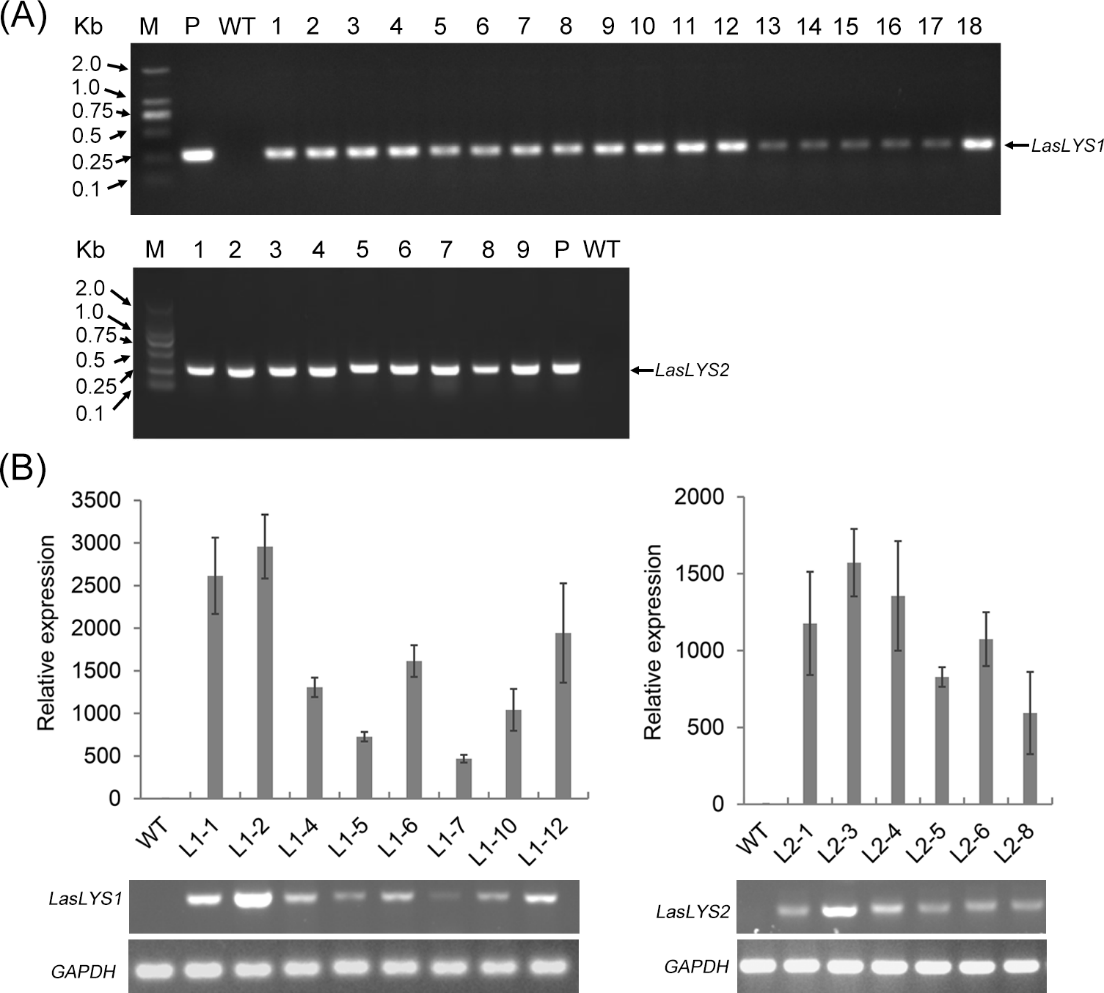


**Figure S4** Determination of transgenic plants expressing *LasLYS1* or *LasLYS2*. **(A)** PCR confirmation of transgenic plants. Using the specific primers shown in Supplementary Data 15, the integration of *LasLYS1* and *LasLYS2* into the genome of transgenic plants was confirmed by PCR. **(B)** qRT-PCR (Up) and RT-PCR (Down) analysis of *LasLYS1* and *LasLYS2* expression in transgenic plants. The citrus *GAPDH* was used as the reference gene for transcript normalization. Vertical bars indicate the standard deviations of the means of three tests.


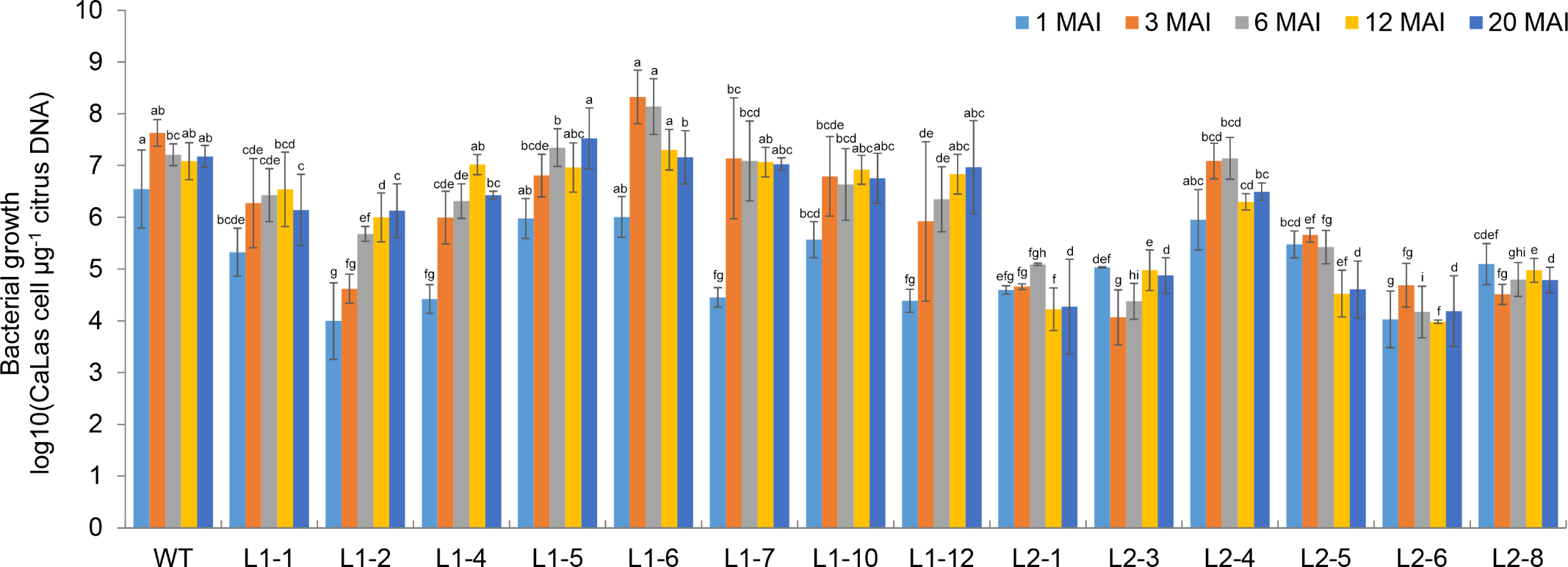


**Figure S5** Characteristics of *Ca*Las growth in the representative transgenic plants. The bacterial populations [log_10_(Las cells μg^-1^ of citrus DNA)] in roots were investigated using qPCR. Values are expressed as means ± standard deviation of three or four plants per line. Different letters on the top of the bars indicate significant differences from the WT control based on a Duncan’s test (*p* < 0.05). WT, wild type; L1-# and L2-#, transgenic plants expressing *LasLYS1* and *LasLYS2*, respectively. MAI indicates month after infection.


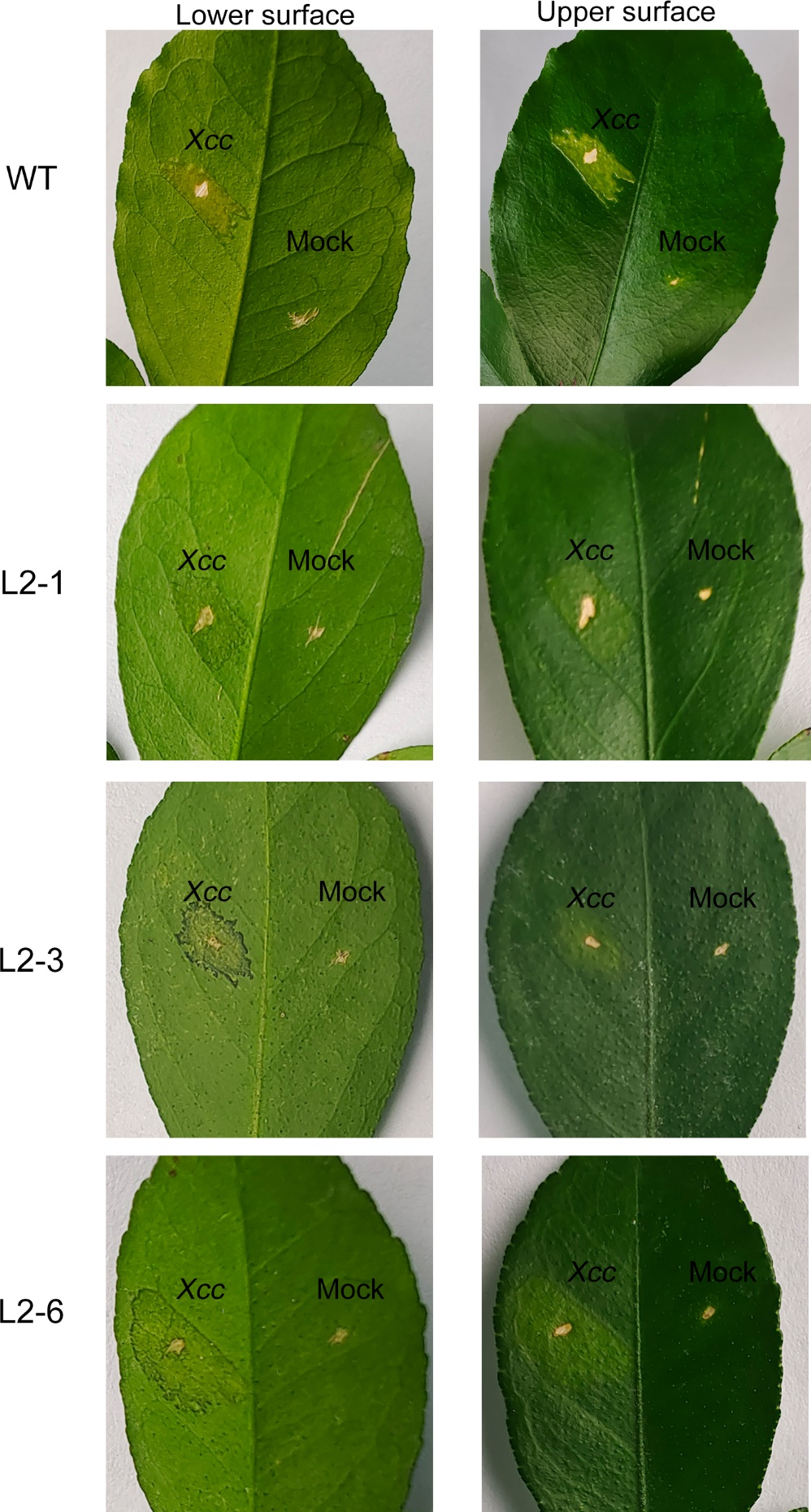


**Figure S6** *In vivo* assay of resistance to citrus canker in transgenic plants. Fully mature healthy leaves of transgenic and wild type plants were infiltrated with *Xanthomonas citri* subsp. *citri* (Xcc) suspensions (1×10^8^ CFU/mL Xcc). Photographs were recorded at 9 days after infiltration. WT, wild type; L2-#, *LasLYS2* transgenic lines. Mock, H_2_O as control. The experiment was independently repeated two times.

**
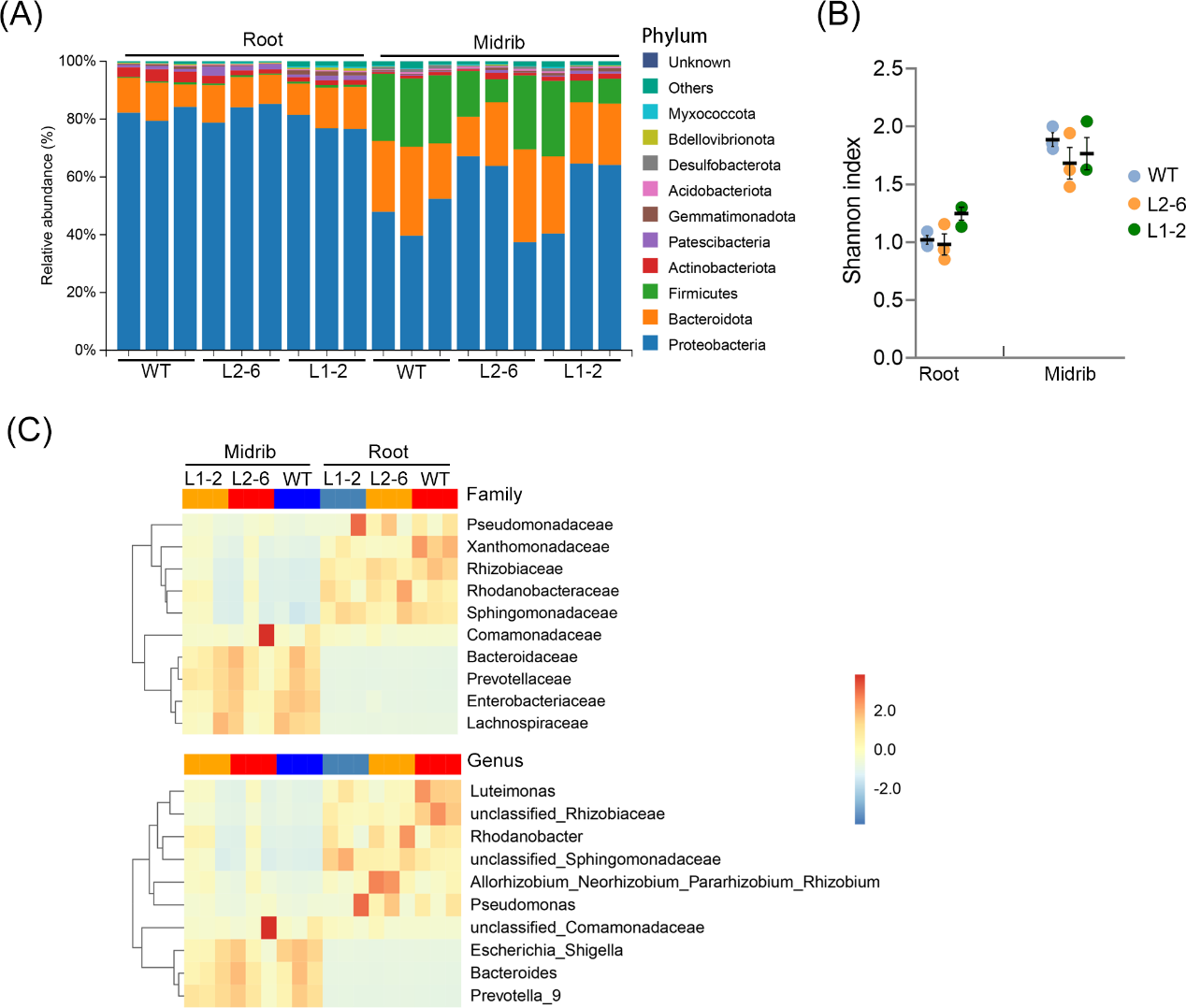
**

**Figure S7** Characteristics of microbiomes in the roots and midribs of transgenic plants. **(A)** Relative abundance of bacterial phyla in the root and midrib microbiomes of L1-2 and L2-6 transgenic plants and WT controls. **(B)** *LasLYS1* and *LasLYS2* expression does not change α-diversity of root and midrib microbiomes compared to WT control (Student’s test; p<0.05; N=3). The plot displays the average Shannon index ± SD. **(C)** Heatmap of relative abundances of bacterial families and genera in L1-2 and L2-6 transgenic plants compared to WT controls.

**
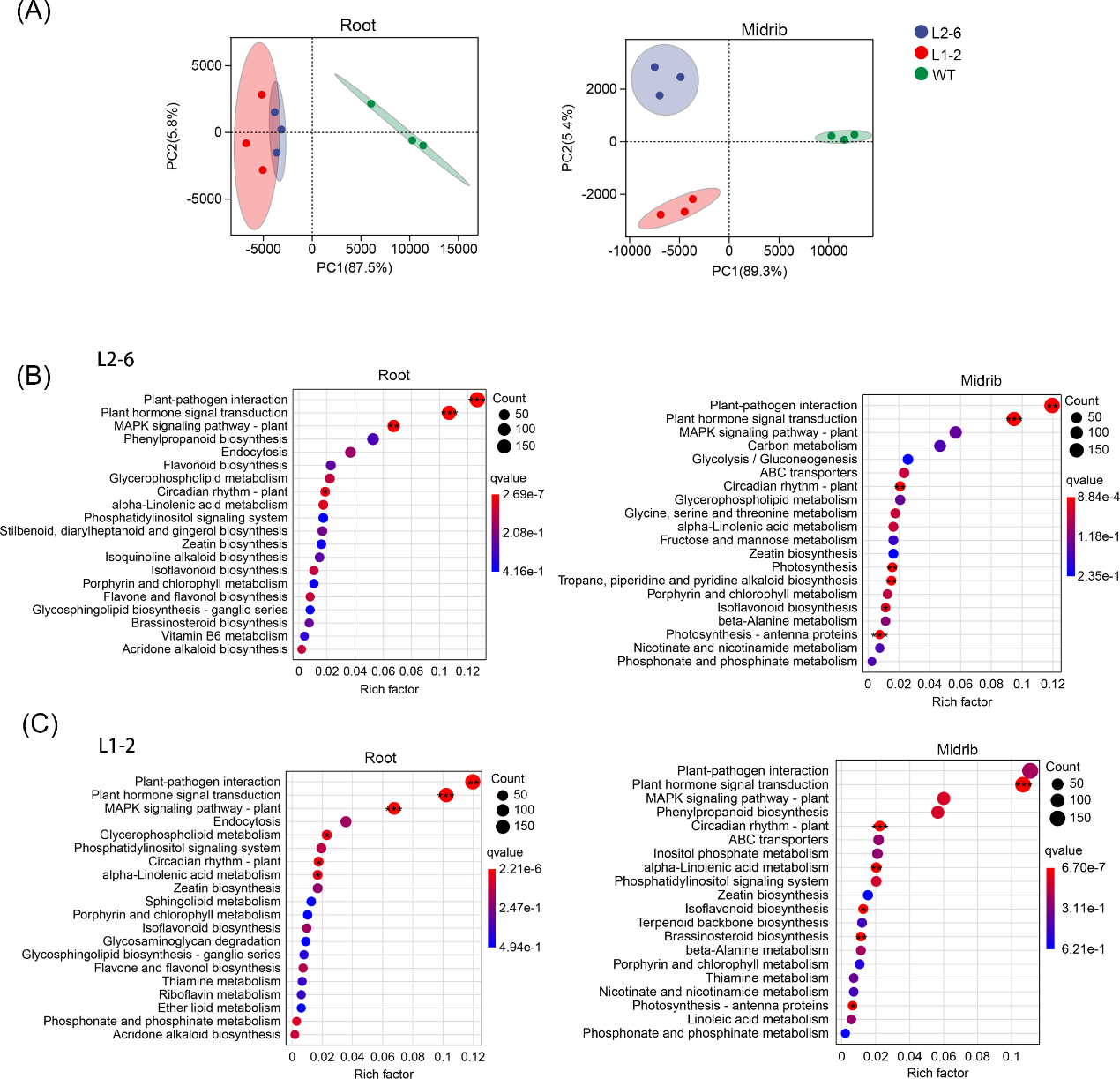
**

**Figure S8** Characteristics of transcriptomic profilings in the roots and midribs of transgenic plants. (A) Principal component analysis (PCA) of gene expression in the roots and midribs of L1-2 and L2-6 transgenic plants and WT controls. (**B**) and (**C**) KEGG pathway enrichment analysis of differentially expressed genes in the roots and midribs of L1-2 and L2-6 transgenic plants. “*”, “**” and “***” indicates significant difference at qvalue < 0.05, 0.01 and 0.001, respectively.


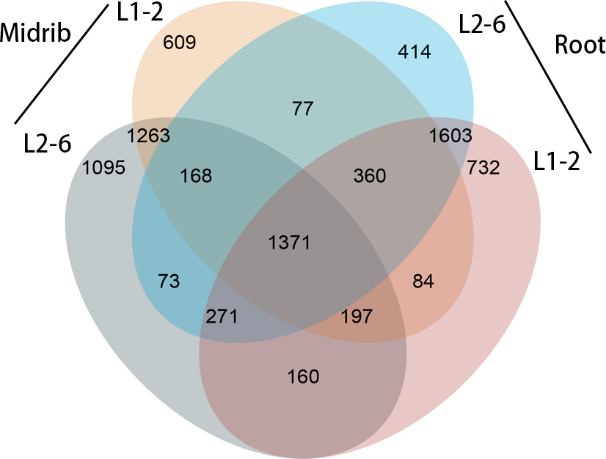


**Figure S9** Overlaps of differentially expressed genes between the roots and midribs of L1-2 and L2-6 transgenic plants.


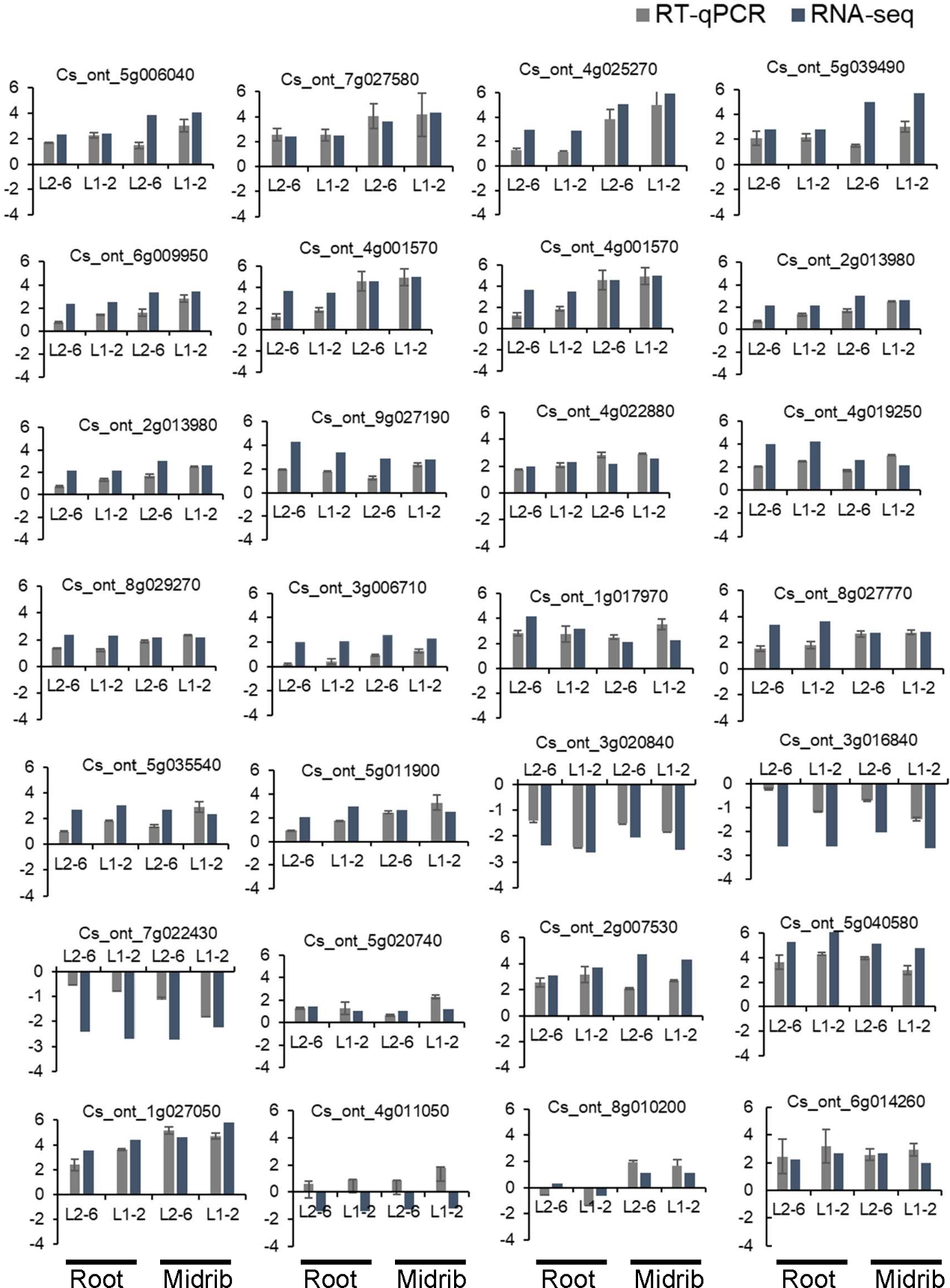


**Figure S10** Validation of 28 differentially expressed genes (DEGs) by RT-qPCR analysis. For these experiments, *GAPDH* gene was used as an internal control. Values are expressed as means ±standard deviation of three biological repeats. L2-6, the transgenic line expressing *LasLYS2*; L1-2, the transgenic line expressing *LasLYS1*; WT, wild type.


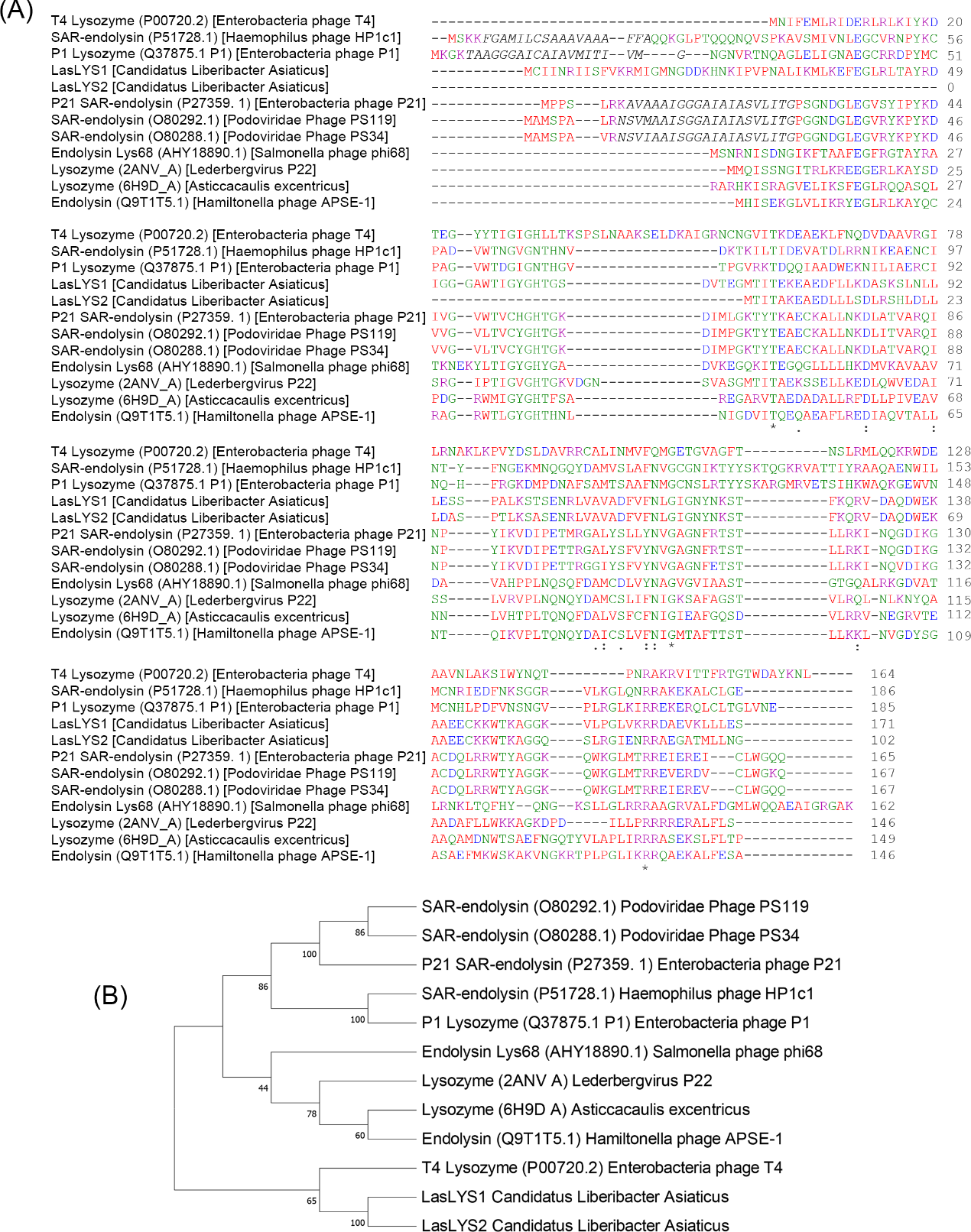


**Figure S11** Functional prediction of LasLYS1 and LasLYS2. The functional domain analysis of LasLYS1 and LasLYS2 was conducted using the NCBI SWISSPROT and PDB database. (**A**) Multiple sequence alignment of LasLYS1 and LasLYS2 with selected homologs. “*”, “:” and “.” indicate a single completely conservative, highly and low similar among residues, respectively. The putative signal-arrest-release (SAR) sequence is highlighted in italics. (**B**) A phylogenetic tree containing LasLYS1 and LasLYS2 and other selected homologs. The reliability of the tree was estimated using 1000 bootstrap replicates and the bootstrap values (%) were shown above branches.


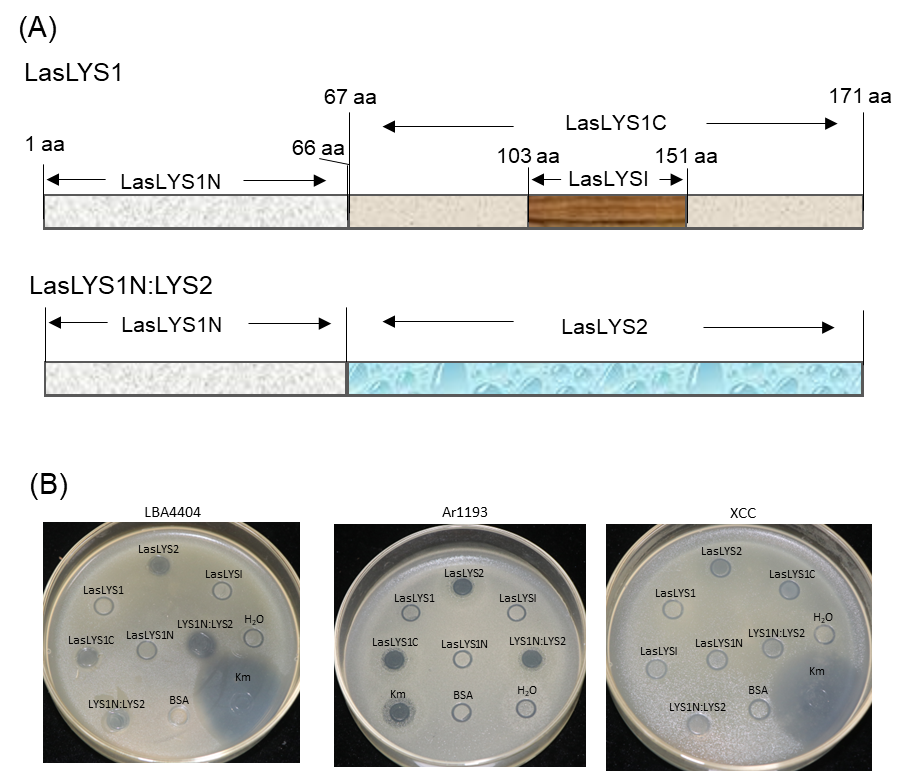


**Figure S12** Bactericidal activity analysis of different LasLYS variants by Oxford cup method. **(A)** Structure diagram of LasLYS variants. **(B)** Observation of bactericidal activity of LasLYS variants. LBA4404, Ar1193 and Xcc strains were tested. 50 μl solution containing 1.5 mg/ml BSA, 1.5 mg/ml LasLYS, 0.5mg/ml Km was added into Oxford cup. For Xcc, 3.0 mg/ml LasLYS was used. After three days of inoculation, the inhibition zone was recorded by photograph.


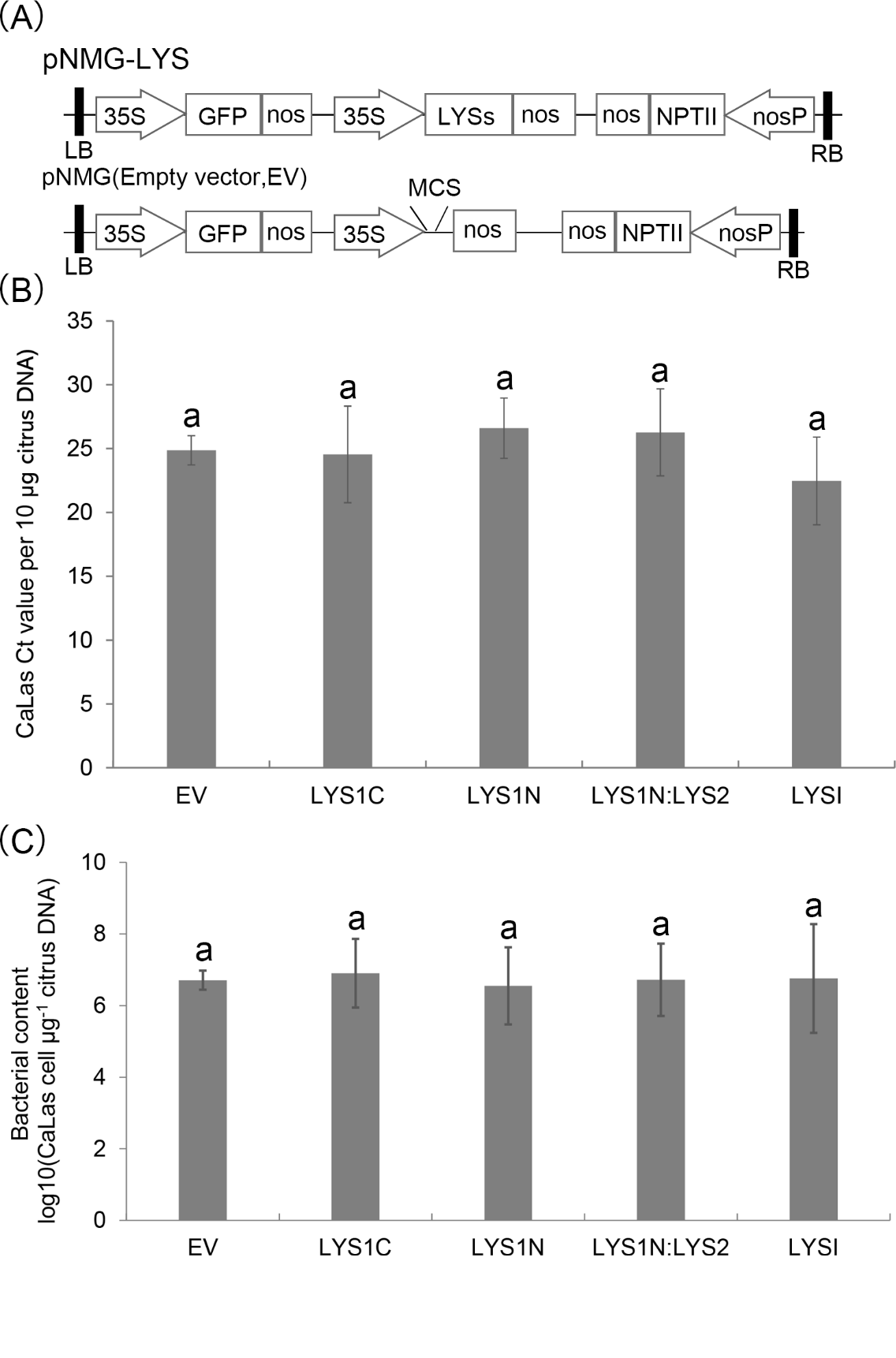


**Figure S13** Determination of *Ca*Las contents in transgenic hair roots at two months after transformation. **(A)** T-DNA structure of plant expression vectors used in the *Rhizobium rhizogenes*-mediated transformation. 35S, CaMV 35S promoter; GFP, Green fluorescent reporter gene; NOS, the nopaline synthase terminator; LYSs, LasLYS endolysin genes; nosP, the promoter of nopaline synthase gene; NPTII, neomycin phosphotransferase genes; LB, left border; RB, right border; MCS, Multiple cloning site. *Ca*Las content was determined by qPCR. *Ca*Las content was expressed by CT value of Las16S gene **(B)** and log10(Las cells μg^-1^ of citrus DNA) **(C)**. Values are expressed as means ± standard deviation of three groups (#1, #2 and #3 in Figure 7). Different letters on the top of the bars represent significant differences from the EV control based on Duncan’s test (*P* < 0.05).


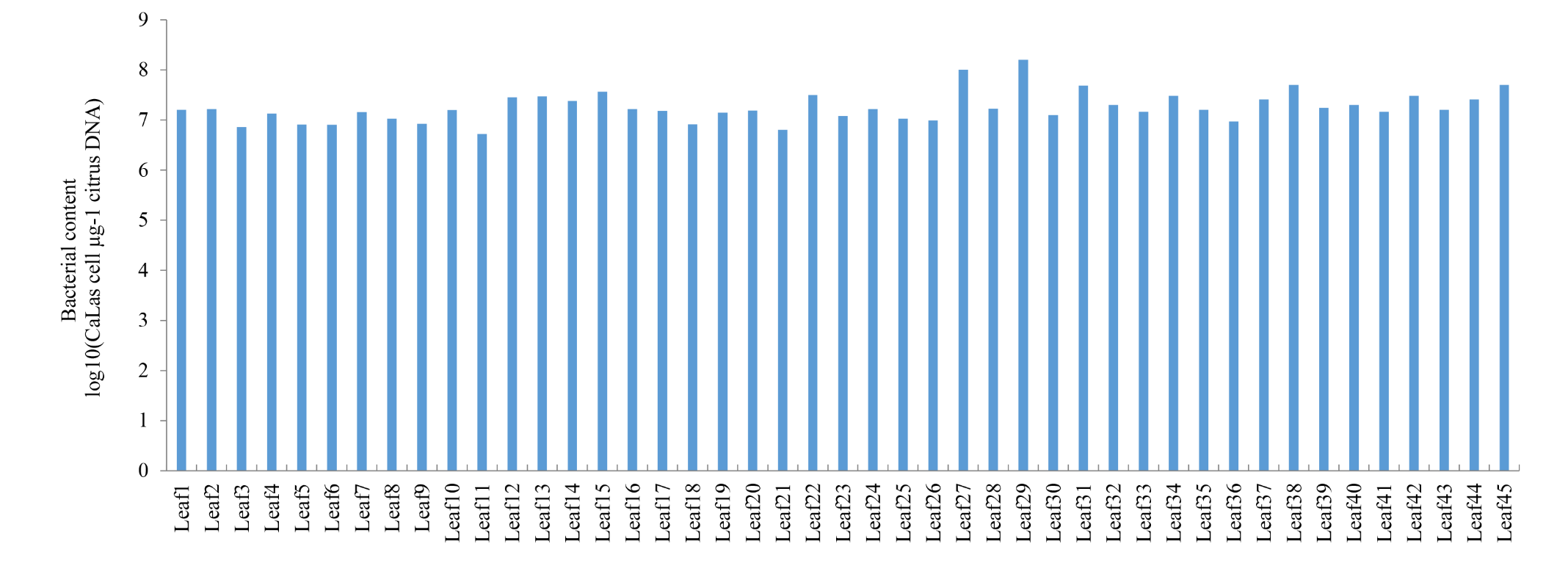


**Figure S14** Determination of CaLas contents in the leaves from *Ca*Las-infected Wanjingcheng oranges for leafdisc grafting. DNA was isolated from the petiole per leaf and CaLas contents [log10(CaLas cells μg^-1^ of citrus DNA)] in DNA sample were determined by qPCR.
